# Time pressure changes how people explore and respond to uncertainty

**DOI:** 10.1038/s41598-022-07901-1

**Published:** 2022-03-08

**Authors:** Charley M. Wu, Eric Schulz, Timothy J. Pleskac, Maarten Speekenbrink

**Affiliations:** 1 Human and Machine Cognition Lab, University of Tübingen, Tübingen, Germany; 2 Center for Adaptive Rationality, Max Planck Institute for Human Development, Berlin, Germany; 3 MPRG Computational Principles of Intelligence, Max Planck Institute for Biological Cybernetics, Tübingen, Germany; 4 Department of Psychology, University of Kansas, Lawrence, KS USA; 5 Department of Experimental Psychology, University College London, London, UK

**Keywords:** Cognitive control, Decision, Learning algorithms, Human behaviour

## Abstract

How does time pressure influence exploration and decision-making? We investigated this question with several four-armed bandit tasks manipulating (within subjects) expected reward, uncertainty, and time pressure (limited vs. unlimited). With limited time, people have less opportunity to perform costly computations, thus shifting the cost-benefit balance of different exploration strategies. Through behavioral, reinforcement learning (RL), reaction time (RT), and evidence accumulation analyses, we show that time pressure changes how people explore and respond to uncertainty. Specifically, participants reduced their uncertainty-directed exploration under time pressure, were less value-directed, and repeated choices more often. Since our analyses relate uncertainty to slower responses and dampened evidence accumulation (i.e., drift rates), this demonstrates a resource-rational shift towards simpler, lower-cost strategies under time pressure. These results shed light on how people adapt their exploration and decision-making strategies to externally imposed cognitive constraints.

## Introduction

We have all experienced the pressure of making decisions under limited time. For instance, choosing what to order at a restaurant while the waiter waits impatiently behind your shoulder. Or deciding which analyses to run as a paper submission deadline looms near. With less time to think, we have less opportunity to perform costly computations. But does time pressure merely make us more noisy as we deal with the speed-accuracy trade-off^1,2^? Or are we able to adapt our decision-making processes, to make the best use of our cognitive resources given external constraints on our computational capacity^3–6^?

Here, we are interested in the cognitive processes involved in navigating the exploration-exploitation dilemma^7–9^, which plays a key role when learning through interactions with the environment, such as in reinforcement learning^10^ (RL) problems. Should you exploit your usual menu option or should you explore something new? The usual option may yield a predictably rewarding outcome, but forgoes the opportunity of learning about other menu items. A new option could lead to either a pleasant or unpleasant surprise, but will likely be informative for future decisions and could improve future outcomes.

Since optimal solutions to the exploration-exploitation dilemma are generally unobtainable^11,12^ except in limiting cases^13–15^ (e.g., infinite time horizons among other assumptions), there is great interest in understanding the strategies that humans use^16,17^. Empirical evidence from a variety of experiments^8,18–21^ and real-world consumer data^22^ suggests people use a mix of two strategies: random and directed exploration. *Random exploration* increases the diversity of choices by adding stochasticity to the agent’s behavioral policy, instead of only maximizing expected value. If you have only ever tried a handful of items on the menu, then you might have an imperfect picture of which options are good. Thus, adding more variability to your choices may give you a better perspective about which options you should value. In contrast, *directed exploration* adds an exploration bonus to each option, proportional to the agent’s level of uncertainty^23^. Rather than simply behaving more randomly, directed exploration is more strategic, prioritizing choices with the highest uncertainty to gain more information^24,25^. Perhaps there is an item on the menu you have never tried before. Directing your exploration to that novel item would be more effective at achieving an information maximization goal than choosing randomly. But since representations of uncertainty need to be factored into the decision-making process, this may be computationally more costly.

### Limiting decision time

We manipulate decision time as a method for imposing external limitations on cognitive resources, to better understand the differential cognitive costs associated with random and directed exploration. With less time “budgeted” for costly computations, resource-rational decision makers^4,5^ might be expected to choose cheaper strategies in order to achieve a better trade-off between the costs of computation and the benefits in terms of reward. One line of research on human decision-making commonly assumes that time pressure causes participants to rely more on “intuitive decision making”^26^, making immediate outcomes more salient^27^, and making people more reliant on fast, recognition-based processes as compared to slower, more analytical processes^28^. Research using formal computational models has also related time pressure to changes in the speed-accuracy trade-off^29^, yielding faster, less accurate decisions, but nevertheless still achieving an efficient rate of rewards^30,31^. However, there is disagreement in the literature about how time pressure changes exploration patterns.

On the one hand, taxing cognitive capacities has been shown to *increase* exploration, producing less consistent and fewer expected value-maximizing decisions^32,33^. Similarly, people and monkeys placed under time pressure become more eager to select uncertain options, independent of outcome value^34,35^. Time pressure has also been linked to making people become more risk-seeking^36–38^, although recent modeling work has challenged the reliability of this shift in risk preferences^33^. Nevertheless, a common thread is that limiting cognitive capacity reduces the scope or detail with which people evaluate different options^39–41^, producing more impulsive decisions or a switch to simpler, heuristic decision-making strategies^42^, both with similar patterns of increased exploration.

On the other hand, time pressure has also been shown to *decrease* exploration, leading to more repeat choice behavior and a reduced preference for uncertain options. Participants under time pressure are more likely to repeat previous actions^43^, even to the detriment of producing more costly errors. This can also be related to a trade-off between reward and policy complexity^44^, where less complex and cheaper-to-encode policies will lead to higher rates of choice perseveration (i.e., repeat choices). Time pressure has also been shown to increase participants’ preferences for a known payoff over an uncertain alternative in the domain of gains^45^, although the inverse was true in the domain of losses. There are also similar findings from description-based gambles, where time pressure can increase risk aversion in the domain of gains^46^.

These divergent results could be interpreted through the lens of early work on coping mechanisms people use when put to the limits of their cognitive abilities^47^. One mechanism is *acceleration*, where information is processed at a faster rate. Combined with lower evidence thresholds, acceleration can lead to more frequently choosing options that would otherwise be ignored, consistent with increased random exploration. Recent work using drift diffusion models has supported this hypothesis by connecting random exploration to lowered evidence thresholds and increased drift rates^48^. Conversely, longer response times have been related to the ability to mentally simulate a greater number of future outcomes^49^, producing more directed exploration but decreased random exploration^50^. Acceleration as a response to time pressure could thus produce a trade-off between different forms of exploration.

Another potential mechanism is *repetition*, where previous actions are repeated or recycled^44,51^, since it may not always be cost effective to simulate any future outcomes at all. This can be related to value-free habits^52^, where not all decisions justify the cognitive costs of using value expectations (both rewards and uncertainty) to select new actions. Whereas you might normally enjoy exploring new restaurants in a new city, limits on decision time, such as an imminent departure at the airport, might motivate you to default to a previously visited restaurant, instead of weighing the alternatives and selecting a new option.

### Goals and scope

**Figure 1:**
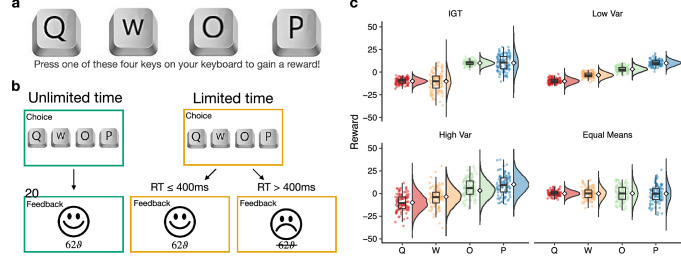
Experimental design. (**a**) Time bandit task, where each option was randomly mapped to the [*Q*, *W*, *O*, *P*] keys on the keyboard, with a different mapping each round. Participants completed 40 rounds (each containing 20 trials), where we manipulated time pressure (**b**) and payoff conditions (**c**) in a crossed, within-subject design. (**b**) In *unlimited time* rounds, participants could take as long as they wanted to make each selection and received positive feedback (happy face) and were shown the value of the acquired payoff for 400 ms (feedback period). In *limited time* rounds, participants were only given 400 ms to make each selection. If they exceeded the time limit, they earned no rewards and received negative feedback (sad face) with the value of the payoff they could have earned crossed out. We used the same feedback period duration of 400ms before the next trial automatically began and participants were shown the choice screen. Inputs during the feedback period had no effect. (**c**) Each payoff condition specifies a normal payoff distribution for each option, with the means and variances described numerically in Table 1. The reward distributions are designed to compare how differences in reward expectations and differences in uncertainty influence choices, where IGT refers to a payoff distribution inspired by the Iowa Gambling Task (see “Methods”). Dots and the Tukey boxplots describe 100 randomly drawn payoffs, while the half violin plots show the generative distribution, with the diamond indicating the mean.

We present a rich experimental setting, where we use a within-subject design manipulating the presence or absence of time pressure to gain insights into the cognitive processes underlying exploration. We use multiple four-armed bandit tasks, where across four payoff conditions (within-subject), we independently manipulate reward expectations and uncertainty across different options (Fig. 1). This allows us to dissociate value-directed and uncertainty-directed choices, where compared to previous studies with two-armed bandit tasks^19,24^, the richer set of options makes efficient exploration more relevant and observable over more trials. Given less decision time, participants can be expected to have less access to costly computations, leading to less value-maximizing choices and more random exploration. Simultaneously, time pressure may limit the capacity for reasoning about the uncertainty of each option, thus leading to less uncertainty directed exploration.

**Table 1:** Payoff conditions.

Payoff conds	Means ($\mu$)	Variances ($\sigma ^2$)
IGT	$[-10,-10,10,10]$	[10, 100, 10, 100]
Low var	$[-10,-\frac{1}{3},\frac{1}{3},10]$	[10, 10, 10, 10]
High var	$[-10,-\frac{1}{3},\frac{1}{3},10]$	[100, 100, 100, 100]
Equal means	[0, 0, 0, 0]	[10, 40, 70, 100]

Means shown are unshifted. In the experiment, a random value between 30 and 60 was added to all rewards of all options, and actual rewards were always positive. IGT refers to payoffs inspired by the Iowa Gambling Task^54,80^.

As predicted, time pressure made participants less sensitive to reward values (more random exploration) and less likely to select options with high relative uncertainty (less directed exploration). We then estimated three hierarchical Bayesian models to understand how expectations of reward and subjective uncertainty influenced choices, reaction times (RTs), and evidence accumulation, which reaffirmed our behavioral analyses, with additional insights into the decision-making and evidence accumulation process. Time pressure diminished uncertainty-directed exploration through several mechanisms: (i) reducing the selection of uncertain options during early trials, (ii) encouraging more aggressive exploitation of known options in later trials, and (iii) heightening the tendency to repeat previous choices.

Our analysis of the RT data revealed how this shift in exploration is related to the computational costs of different exploration strategies. High reward expectations corresponded to faster choices, while high uncertainty (both relative and total) were associated with slower choices. Under time pressure, participants selected highly rewarding options even faster, but slowed down less when selecting highly uncertain options—independent of having faster choices in general. These changes in RT can be linked to the evidence accumulation process. While time pressure did not change how reward expectations influenced evidence accumulation (faster choices were due to lower decision-thresholds), it reduced the extent that relative uncertainty dampened the rate of evidence accumulation.

Our findings indicate that time pressure selectively impacts how uncertainty is integrated into decisions. Put under time pressure, people are less influenced by uncertainty, less value-directed, and more likely to repeat previous choices. This is a simpler strategy and comes at lower costs, representing a potentially resource-rational adaptation to time pressure. These results enrich our understanding of human exploration strategies under changing task demands, providing insights into the cognitive costs of reasoning about and acting on uncertainty.

## Results

We conducted an online experiment on MTurk ($n=99$; 36 female; $M_{\text {age}}$ = 34.82; $SD_{\text {age}}$ = 10.1)) to study how time pressure influences exploration behavior (Fig. 1). Our “Time Bandit” experiment employed repeated four-armed bandit tasks, where we independently manipulated expected reward and uncertainty across four payoff conditions (Fig. 1c; Table 1), along with time pressure (limited vs. unlimited time). This allowed us to disentangle how relative differences in reward expectations and uncertainty influence choices, and how time pressure modulates this influence, in a single within-subject design (see “Methods”).

### Behavioral analyses

**Figure 2:**
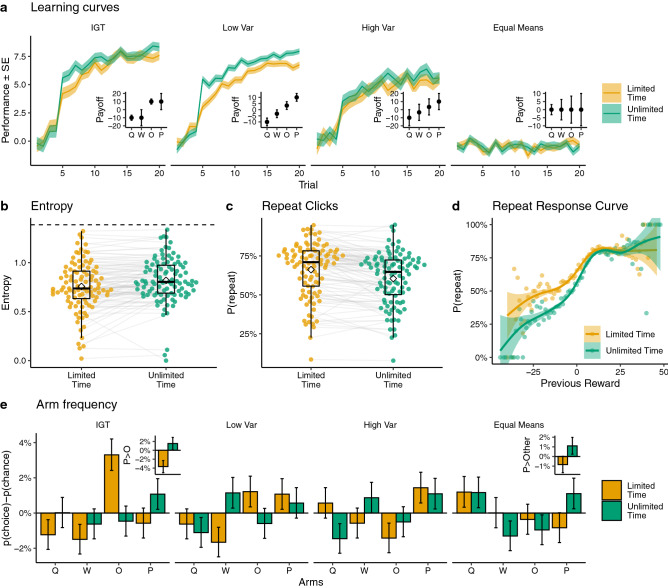
Behavioral results. (**a**) Learning curves depicting average participant performance (lines) ± standard error of the mean (ribbons) over trials (using unshifted rewards), faceted by payoff condition. The inset figures show the expected reward ± standard deviation of each payoff condition for reference. IGT refers to payoffs inspired by the Iowa Gambling Task (see “Methods”). (**b**) Choice entropy in each round, where higher entropy corresponds to more diverse choices and dotted lines indicate random chance (i.e., playing each arm with uniform probability). Each connected dot represents a participant, and overlaid are Tukey boxplots with the diamond indicating the group mean. (**c**) The proportion of repeat clicks across time conditions, where each connected dot is a single participant, with overlaid Tukey boxplots and the diamond indicating the group mean. (**d**) Repeat choices as a function of the previous (unshifted) reward value. Each dot is the aggregate mean, and lines represent a locally smoothed Generalized Additive Model regression estimate, with the ribbon indicating the 95% confidence interval. (**e**) Aggregate choice proportions (normalized for chance) for each option, mapped to the canonical ordering shown in panel a (inset). Error bars indicate the 95% CI. The inset plots show a preference for the ‘P’ option over the ‘O’ option in the IGT condition, and a preference for the ‘P’ option over all others in the Equal Means condition. See Fig. S1 for a Bayesian mixed effects regression of the behavioral results, and Figs. S2–S4 for additional behavioral analyses.

To analyze the influence of time pressure and payoff conditions on performance and choice behavior, we constructed a series of Bayesian mixed-effects regression models. Specifically, we estimated how average rewards (Fig. 2a), the entropy of choices (Fig. 2b), the number of repeat choices (Fig. 2c), and the probability of making a repeat choice conditioned on payoff (Fig. 2e), were influenced by time pressure and payoff conditions, whilst also accounting for individual differences in the random effects structure. This allows us to describe the influence of either time pressure or payoff conditions in terms of the estimated marginal means ($\Delta _{EMM}$), which uses contrast analyses to quantify differences in the dependent variable marginalized over the other independent variables. For instance, examining how average rewards were influenced by time pressure, marginalized over the four payoff conditions, or vice versa. The raw posterior estimates are provided in Table S1 and visualized in Fig. S1, while Fig. S2 provides the raw data separated by payoff condition.

### Learning curves

Looking first at average reward (Fig 2a), we find that time pressure played a reliable role in reducing rewards ($\Delta _{EMM} = -0.19$ $[-0.29, -0.10]$; all Bayesian estimates include the 95% Highest Density Interval in square brackets). There was also substantial variation across payoff conditions. Participants performed better in the IGT-like condition than in the Low Var condition ($\Delta _{EMM} = 0.12$ [0.04, 0.20]). We see an even larger difference when comparing the Low Var and High Var conditions ($\Delta _{EMM} = 0.24$ [0.15, 0.33]), where despite having the same expected rewards for each option, participants performed substantially better with lower variance. Lastly, participants performed better in High Var than in the Equal Means condition ($\Delta _{EMM} = 1.02$ [0.93, 1.11]), which is intuitive since improvement is not possible if all arms have the same expected reward.

### Entropy and repeat choices

Next, we assessed the overall diversity of choices by calculating the Shannon entropy^53^ of choice distributions in each round (Fig 2b). Participants made less diverse and lower entropy choices under limited time ($\Delta _{EMM} = -0.12$ $[-0.23 -0.01]$). This provides initial evidence for reduced exploration under time pressure. We also find largely overlapping entropy levels among the different payoff conditions, but with Equal Means having the most diverse choices (compared against High Var: $\Delta _{EMM} = 0.34$ [0.27, 0.42]). This suggests that in the face of indiscernible differences in reward expectations, participants increased their exploration.

Additionally, we modeled the number of repeat choices in each round as a measure of sequential dependency between choices (Fig 2c). We used a Binomial regression, modeling the number of repeats as the result of 19 independent Bernoulli trials, since the first choice cannot be a repeat by definition. Participants made overwhelmingly more repeat choices under time pressure (Odds Ratio (OR): $\Delta _{EMM} = 1.40$ [1.22, 1.58]). While we see relatively small variation across payoff conditions, the Low Var condition had more repeats than the High Var condition (OR: $\Delta _{EMM} = 1.34$ [1.23, 1.47]; see Fig. S1c), perhaps because participants were able to more quickly identify and exploit the highest rewarding arm with less variance in observed outcomes.

Lastly, we also included a variant of the repeat choice model, which included the (unshifted) value of the previous reward as an additional predictor (Fig. 2d). Here, we modeled the probability of each choice (after the first trial) being a repeat using logistic regression. We find the same influence of the experimental manipulations on repeat behavior as above (see Table S1), but also find an interaction between time pressure and previous reward value. Participants were more likely to repeat a choice with higher rewards in unlimited time (OR $=1.24$ [1.19, 1.29]). Put differently, time pressure reduced participants’ sensitivity to reward value in their repeat behavior, as evidenced by the flatter response curve in Fig. 2d.

### Choice patterns

Figure 2e visualizes the aggregate choice proportions to get a better sense of patterns related to reward expectations and uncertainty. These bars indicate the aggregate choice frequency of each option relative to chance, where bars above zero indicate the option was chosen more frequently, and bars below zero indicate the option was chosen less frequently. The difference between the orange and green bars illustrates the differences in choice behavior as a function of time pressure. We aggregate the data using the canonical mapping of reward distributions (see inset plots in Fig. 2a for reference) to the [*Q*, *W*, *O*, *P*] keys, although the keys were randomly mapped in each round for participants. To provide statistical support for choice differences, we use Bayesian mixed-effects logistic regression to model how time pressure influenced the probability of choosing a given option. We focus on two informative cases.

In the IGT condition (named for mimicking the structure of the so-called Iowa Gambling Task^54^), there were two high reward and two low reward options, with each pair having either a low or high variance. We focused on the two high reward options (indicated as ‘O’ and ‘P’ in Fig. 2e), and modeled whether time pressure influenced the likelihood of choosing the riskier, high variance option ‘P’ over the safer low variance option ‘O’, as a simple test of how decision time can influence the role of relative uncertainty (see Fig. 2e inset for the raw data). We found that overall, participants chose the high variance option (‘P’) more frequently in unlimited time (Odds Ratio: OR $=1.11$ [0.80, 1.53]; Table S2), although the estimates overlapped with chance (OR $=1$). However, there was also an interaction with round number, where the difference between time conditions widened over successive rounds. Participants in the unlimited time condition increased their likelihood of selecting the high variance option over rounds (OR $=1.39$ [1.23, 1.57]). This effect tended towards the opposite direction for limited time rounds, where participants selected the high variance option less frequently over rounds (OR $=0.83$ [0.68, 1.02]).

We find the clearest differences arising from the time-pressure manipulation in the Equal Means condition (Fig. 2e inset), where compared against all other options, participants were more likely to select the highest variance option (‘P’) in the unlimited time condition (OR $= 1.44$ [1.12, 1.86]; Table S2). This illustrates a clear shift in preferences away from uncertain options when time pressure is introduced. Whereas participants tend to be risk-seeking and choose more uncertain options under unlimited time, they become more risk-averse and choose them less often under time pressure.

### Interim discussion

Altogether, we find behavioral evidence that time pressure reduced exploration. There were less diverse and more repeat choices, which ultimately resulted in lower reward outcomes. From these analyses, we find two important behavioral signatures of the underlying cognitive processes that produced this shift in exploration. First, time pressure reduced participants’ sensitivity to reward values in repeating previous choices, making them more likely to repeat a low-reward choice (Fig. 2d). Second, participants were less likely to select options with higher relative uncertainty under time pressure (Fig. 2e). In the next section, we employ model-based analyses, which use RL models to explicitly track expected reward and uncertainty estimates. We then use these estimates to model choice behavior, reaction times, and evidence accumulation (i.e., drift rate).

### Model-based analyses

To model learning and decision making in our task, we use a *Bayesian mean tracker* (BMT) as an RL model for estimating expected rewards and associated uncertainties, which are then updated based on prediction errors (see “Methods”). The BMT is a special case of the Kalman filter, which assumes time-invariant reward distributions (as was the case in our experiment). The BMT provides a Bayesian analogue^55^ to the classic Rescorla-Wagner^56^ model of associative learning, and has described human behavior in a variety of multi-armed bandit and decision-making tasks^19,20,24,57–59^.

**Figure 3:**
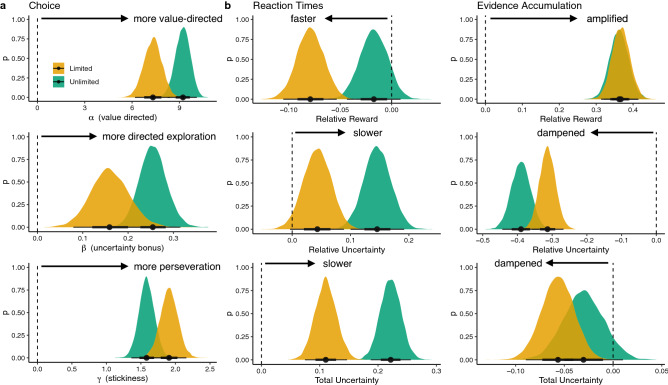
Posterior estimates of model-based analyses. (**a**) Hierarchical softmax model. Expected rewards and uncertainties were regressed onto choice probability (Eqs. 1–2). The top row shows the value-directed component ($\alpha$), the middle row shows the uncertainty bonus ($\beta$), and the bottom row shows stickiness ($\gamma$). (**b**) Hierarchical RT model. The influence of relative reward (top), relative uncertainty (middle), and total uncertainty (bottom) on RTs. (**c**) LBA drift regression. Relative reward (top), relative uncertainty (middle), and total uncertainty (bottom) were used to predict the drift rate of an LBA. In all plots, the vertical dashed line indicates an effect of 0, while the black dot indicates the mean effect and confidence intervals show the 66% (thick) and 95% (thin) highest density interval.

**Figure 4:**
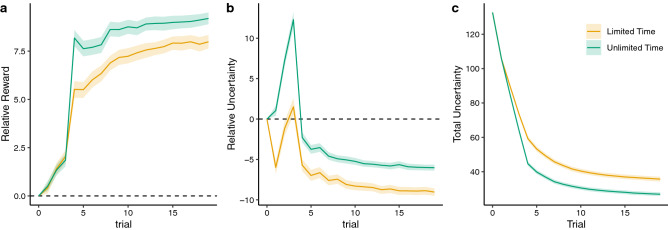
BMT predictions about the chosen option simulated for all participants (see Fig. S5 for more detailed plots, separated by payoff condition). Lines indicate group means, with ribbons showing the 95% CI. (**a**) Relative reward shows the difference between the posterior mean of the chosen option and the average posterior mean of the unchosen options. Relative reward is always valued positively (dashed line indicates 0). (**b**) Relative uncertainty shows the difference between the posterior uncertainty (stdev) of the chosen option and the average posterior uncertainty of the unchosen options. The early upticks indicates uncertainty-directed exploration (substantially less in limited time), followed by exploitation as this value decays below zero (dashed line). (**c**) Total uncertainty (average stdev) decays monotonically, with a faster decline in unlimited time due to more uncertainty directed exploration.

We generated predictions from the BMT using participant choices and reward observations at each trial *t* to compute posterior distributions of the average reward of the options, and then using these as prior predictive distributions at trial $t+1$. These prior predictive distributions are all normally distributed, and we used the mean and standard deviation as measures of predicted reward and the associated uncertainty, respectively (see Figs. 4 and S5), which we use to conduct three model-based analyses predicting choices, reaction times, and evidence accumulation (Fig. 3).

### Choices

In our first analysis, we assessed how reward expectations and uncertainty estimates influenced the likelihood of an option being chosen on each trial. We applied hierarchical Bayesian inference to estimate the parameters of a softmax policy (see “Methods”), under the assumption that a participant’s choice on each trial is influenced by both the predicted mean and uncertainty of an option. Each participant’s parameters are assumed to be jointly normally distributed and assumed to interact with time pressure. The probability of choosing option *j* on trial *t* is a softmax function of its decision value $Q_{j,t}$:

1 $$\begin{aligned} P(C_{t}=j) = \frac{\exp (Q_{j,t})}{\sum _{k=1}^4 \exp (Q_{k,t})}. \end{aligned}$$

The decision value $Q_{j,t}$ is a function of the prior predictive mean $m_{j,t}$ and uncertainty $\sqrt{v_{j,t}}$ (standard deviation) of each option according to the BMT, with an additional stickiness bonus for the most recently chosen option ($\delta _{j,t-1}=1$ if option *j* was chosen on trial $t-1$; see “Methods”):

2 $$\begin{aligned} Q_{j,t} = \alpha (m_{j,t} + \beta \sqrt{v_{j,t}}) + \gamma \delta _{j,t-1}. \end{aligned}$$

We computed hierarchical Bayesian estimates for the value-directed component $\alpha$ (factoring in both rewards and uncertainty), the uncertainty bonus $\beta$ (governing the trade-off between exploitation and exploration), and the stickiness bonus $\gamma$, including interactions with the time pressure manipulation (limited vs. unlimited). Larger $\alpha$ estimates indicate more value-directed choices, whereas lower $\alpha$ suggest more random choices, which are not explainable by reward expectations or uncertainty estimates (i.e., random exploration). More positive $\beta$ estimates indicate a higher level of uncertainty-directed exploration. Higher estimates of $\gamma$ indicate more perseveration in choice behavior, with more frequent repetitions of previous choices. Figure  3a shows the posterior estimates of the model (see Figs. S7–S8 for comparison to alternative models).

We find less *value-directed* choice behavior under time pressure ($\alpha _{\text {Unlimited}} - \alpha _{\text {Limited}}=1.90$ [1.24, 2.56]), with positive estimates in both conditions ($\alpha _{\text {Unlimited}}=9.21$ [8.31, 10.1]; $\alpha _{\text {Limited}}=7.31$ [6.23, 8.44]). This pattern can be seen in the raw BMT predictions (Fig. 4a), where chosen options had both higher relative reward expectations and relative uncertainty in unlimited time. By definition, the inverse of the value-directed component defines the level of random exploration, with the interpretation that participants’ choices were less predictable and more random when given less time to deliberate (limited time). This may seem at odds with the behavioral results showing reduced entropy under time pressure, but the lack of correlation between $\alpha$ and choice entropy under time limitations (see Fig. S6b) suggests that participants consistently chose non-value maximizing options (i.e., repeating low-value choices; Fig. 2e). In contrast, $\alpha$ estimates were correlated with higher average rewards in both conditions (see Fig. S6a).

Time pressure also reduced *uncertainty-directed exploration* ($\beta _{\text {Unlimited}} - \beta _{\text {Limited}}=0.09$ [0.04, 0.15]), with positive estimates in both conditions ($\beta _{\text {Unlimited}} = 0.26$ [0.20, 0.32]; $\beta _{\text {Limited}} = 0.16$ [0.08, 0.24]). Figure 4b provides additional clarity about this result. Participants with unlimited time experienced an early uptick in selecting relatively uncertain options around trial 3, suggestive of an “exploration phase”. Afterwards, there was a gradual shift towards exploitation, indicated by the monotonic decay of the relative uncertainty of chosen options, indicating an increasing preference for relatively less uncertain options. Under time pressure, there is a similar trend, yet the early exploration phase has almost vanished (the relative uncertainty of the chosen option on trial 3 is indistinguishable from 0: $t(98)=0.9$, $p=0.387$, $d=0.1$, $BF=0.16$) and later trials are associated with more strongly negative relative uncertainty. Thus, a reduced exploration bonus under time pressure appears to be a combination of less exploration in early trials, and more aggressive exploitation in later trials, which is also apparent in the higher levels of total uncertainty during limited time rounds (Fig. 4c). This reduction in directed exploration may also be related to the lower overall performance under time pressure, since higher $\beta$ estimates in the limited time condition were associated with higher rewards (see Fig. S6a).

In addition to these changes in exploration, time pressure increased the *stickiness* of choices ($\gamma _{\text {Unlimited}} - \gamma _{\text {Limited}}=-0.32$ $[-0.46,-0.19]$), with positive estimates in both conditions ($\gamma _{\text {Unlimited}}=1.58$ [1.36, 1.80]; $\gamma _{\text {Limited}}=1.91$ [1.64, 2015]). This increase in choice perseveration is consistent with the reduced entropy and higher repeat choice probabilities found in the behavioral data, but estimates of $\gamma$ were unrelated to average reward (see Fig. S6).

Overall, time pressure reduced the value-directedness of choices, reduced uncertainty-directed exploration, and increased the stickiness of choices. We now turn to modeling reaction times (RTs) to better understand how reward expectations and uncertainty influenced the speed of decisions.

### Reaction time

Our second analysis looked at how RTs (see Fig. S3 for raw RT analysis) were influenced by expectations of rewards and estimated uncertainties. We first computed the relative reward and relative uncertainties of the BMT predictions using the difference between the chosen option and the average of the unchosen options on each trial. Thus, positive values indicate that the expected reward or uncertainty were larger than the mean of the unchosen options. We also computed total uncertainty, based on the sum of uncertainty estimates across the four options on any given trial. We then regressed relative mean, relative uncertainty, total uncertainty, and round number onto log-transformed RTs in a Bayesian mixed effects regression (see “Methods”).

The resulting posterior parameter estimates (Fig. 3b) indicate that higher relative reward expectations produced faster choices under limited time ($b_{\text {Limited}}=-0.08$ $[-0.11,-0.05]$), but with a weaker effect under unlimited time ($b_{\text {Unlimited}}=-0.02$ $[-0.04,0.01]$) that overlapped with zero. In contrast, both relative and total uncertainty slowed down choices (relative uncertainty: $b_{\text {Unlimited}}=0.15$ [0.10, 0.19]; total uncertainty: $b_{\text {Unlimited}}=0.22$ [0.19, 0.26]), with the latter having a larger effect. In both cases, this uncertainty-related slowdown was reliably less pronounced when placed under time pressure (relative uncertainty: $b_{\text {unlimited}} - b_{\text {limited}}=-0.10$ $[-0.14,-0.06]$; total uncertainty: $b_{\text {unlimited}} - b_{\text {limited}}=-0.11$ $[-0.14,-0.08]$). Thus, higher reward expectations made people faster, whereas uncertainty (both relative and total) slowed them down. Both effects were less pronounced under time pressure.

There was also a notable interaction between predictors (see Fig. S9 for the full model and Figs. S10–S11 for interaction plots). While high relative reward expectations generally sped up choices, this pattern was inverted when high rewards were also accompanied by high relative uncertainty, with participants slowing down instead of speeding up ($b=0.04$ [0.001, 0.08]; no difference between time conditions). Thus, certainty about high rewards produced rapid decisions, whereas uncertainty about high rewards produced slower choices.

Overall, more exploitative choices (with higher relative reward expectations) were faster, while more explorative choices (with both higher relative uncertainty or higher total uncertainty) were slower. This differs from previous findings using two-armed bandits^19^, in which higher relative uncertainty was related to faster decisions. Here, we find that uncertainty is not just a bonus that adds to the decision signal, making choices easier and faster. Rather, grappling with uncertainty takes time.

### Evidence accumulation

In our third analysis, we used a Linear Ballistic Accumulator^60,61^ (LBA) to model choices and RTs simultaneously (see “Methods”). This model assumes that choices are the result of an evidence accumulation process, where evidence for each option accumulates as a function of drift rate, which is independently estimated for each option. Whichever option first exceeds the decision threshold is chosen. The interplay between the drift rate and evidence threshold captures how participants trade response speed for accuracy, with higher thresholds requiring more evidence and producing more value maximizing choices, yet slower responses. Thus, we can use the LBA to separate out how time pressure impacts evidence accumulation in terms of the rate of evidence accumulation and the amount of evidence collected.

Consistent with the need to arrive at decisions more quickly, we observed both lower relative evidence thresholds *k* ($t(98)=-5.2$, $p<0.001$, $d=0.5$, $BF>100$; Fig. S12) and higher mean drift rates ($t(98)=7.1$, $p<0.001$, $d=0.7$, $BF>100$) under time pressure. This suggests an accelerated processing of information (faster drift rates), which is also more prone to errors (lower threshold). The maximum pairwise difference between drift rates was also larger under limited time ($t(98)=6.0$, $p<0.001$, $d=0.6$, $BF>100$), suggesting larger separation between different options, which is also related to lower choice entropy and more frequent repeat choices (see Fig. S13). Additionally, participants had shorter non-decision times $\tau$ ($t(98)=-4.6$, $p<0.001$, $d=0.5$, $BF>100$) and less maximum starting evidence *A* ($t(98)=-7.8$, $p<0.001$, $d=0.8$, $BF>100$), when placed under time pressure. All parameters where strongly correlated across time conditions (Kendall rank correlations; all $r_\tau >0.40$; $BF>100$; Fig. S12), are recoverable (Fig. S14), and can be used to simulate realistic choice and RT patterns (Fig. S15). Thus, our LBA results confirm the intuition that participants reached faster decisions at lower evidence thresholds when time limitations were imposed, but they also accumulated evidence faster and with larger separation between options.

In a final step, we sought to better understand how expectations of reward and uncertainty influence the evidence accumulation process and how time pressure may impact this relationship. Thus, we regressed the BMT predictions of relative expected reward, relative uncertainty, and total uncertainty for each option onto its estimated drift rate using a Bayesian mixed effects regression. Note that the LBA parameters are estimated on each round, thus the BMT predictions are averaged over trials, but nevertheless capture differences in the trajectory of learning and the independent manipulations of expected rewards and uncertainty in the four payoff conditions.

The result of this analysis (Fig. 3c) revealed that higher relative reward expectations amplified evidence accumulation equally for limited and unlimited time ($b=0.36$ [0.31, 0.41]; no interaction with time pressure: $b_{\text {Unlimited - Limited}}=-0.005$ $[-0.061,0.051]$). Thus, options with higher relative reward expectations were more likely to be chosen and with faster decision times. Conversely, relative uncertainty (specific to each option) had a negative effect on drift rate, thus dampening evidence accumulation ($b_{\text {Unlimited}}=-0.39$ $[-0.44,-0.34]$), with a reliably smaller effect under time pressure ($b_{\text {Limited}}=-0.31$ $[-0.36,-0.27]$; $b_{\text {Unlimited - Limited}}=-0.08$ $[-0.14,-0.01]$). Lastly, total uncertainty (computed across all options) also dampened evidence accumulation in limited time rounds ($b_{\text {Limited}}=-0.06$ $[-0.09,-0.03]$), but did not produce a reliable effect in unlimited time ($b_{\text {Unlimited}}=-0.03$ $[-0.07,0.01]$). Thus, rewards increased evidence accumulation, while uncertainty (in general) slowed down evidence accumulation.

The main interaction between predictors (see Fig. S16 for the full model and Figs. S17–S18 for interaction plots), was that the effect of total uncertainty could be inverted depending on relative reward (no interaction with time pressure: $b=-0.08$ $[-0.14,-0.02]$; Fig. S17g) and relative uncertainty ($b_{\text {Limited}}=-0.15$ $[-0.18,-0.12]$; $b_{\text {Unlimited}}=-0.09$ $[-0.13,-0.06]$; Fig. S17h). Total uncertainty amplified evidence accumulation when the stakes were low (low relative rewards or low relative uncertainty), but dampened evidence accumulation instead when the stakes were high (high relative rewards or relative uncertainty). Since total uncertainty is the same across all options, amplified evidence accumulation under low stakes corresponds to faster, more random choices, consistent with little benefit from increased deliberation in these settings. Conversely, dampened evidence accumulation under high stakes corresponds to slower, and more reward- or uncertainty-directed choices.

Overall, we find that reward-modulated increases in evidence accumulation were unaffected by time pressure. However, uncertainty-driven decreases in evidence accumulation were less pronounced under time pressure, with drift rates less influenced by uncertain options. We also found an influence of high total uncertainty, which was modulated by expectations of rewards and relative uncertainty. When more was at stake, total uncertainty dampened drift rates and produced slower decisions. But when relative differences in reward expectations were minor, higher total uncertainty amplified drift and produced faster decisions.

## Discussion

How is exploration and decision-making constrained by cognitive limitations imposed through time pressure? We investigated this question using several variants of a four-armed bandit task, designed to independently manipulate differences in reward expectations and uncertainty. We then used a time pressure manipulation to either give participants unlimited decision time or to limit decision time to less than 400 ms for each choice. Both payoff and time pressure manipulations were conducted within-subjects, allowing us to use hierarchical modeling to achieve a high level of detail into the interplay between learning strategies and cognitive limitations imposed by time pressure.

Our behavioral results show that time pressure induced participants to earn fewer rewards, made them less sensitive to reward values in their repeat choice behavior, and less likely to select options associated with higher uncertainty. We then used RL models to analyze how reward expectations and uncertainty affected choices, RTs, and the rate of evidence accumulation.

High reward expectations made participants more likely to select options, producing faster RTs for such exploitative choices, and amplifying the rate of evidence accumulation. Adding time pressure reduced the value-directedness of choices, but increased their tendency to speed up when choosing options with high relative reward expectations (i.e., exploitation), and made them more likely to repeat previous choices.

In contrast, while uncertainty also made participants more likely to select options, choices with higher relative (and to some extent total uncertainty) were associated with slower choices and reduced evidence accumulation rates. Adding time pressure reduced uncertainty-directed exploration in choice behavior and also reduced the influence of uncertainty on RTs. This is consistent with the notion that uncertainty takes time to process and deploy strategically. Without the necessary time to grapple with uncertainty, participants shifted to exploiting known options and repeating previous choices, rather than integrating the value of exploring uncertain options.

Similar reductions in directed exploration have also been observed when participants were placed under working memory load^62^. The resulting behavior may thus be seen as a resource-rational^4,5^ adaptation to externally imposed limitations on cognitive resources, consistent with other findings showing that people are sensitive to the cost-benefit tradeoffs of different learning strategies^63,64^. Indeed, the interactions of our LBA model (Fig. S17g,h) suggest that people are sensitive to the cost-benefit trade-off of increased deliberation, producing faster more random decisions when the stakes are low, but slowing down and deliberating longer when the stakes are high. Future research should examine the underlying mechanisms of the arbitration between strategies and the neural locus of cognitive control.

### Limitations and extensions

One limitation is that we only account for how time pressure influences exploration strategies, but not for changes in learning. Time pressure might not only change which computations we engage in when deciding how to explore or exploit, but it might also influence the richness of the representations we form during learning or the extent to which these representations are updated in response to new information. Indeed, previous work in economics has shown a reduced efficacy of training^65^. However, our use of Bayesian RL in modeling choices and RTs may not be able to differentiate between these hypotheses, although similar models in related tasks have been used to predict directly elicited participant judgments about reward expectations and confidence^66–70^. Future studies may consider modeling not only choices and RTs, but also participant judgments about future outcomes in a similar time pressure manipulation.

Our current results also only examined uncertainty about reward expectations. However, there exist several alternative measures of uncertainty such as confidence^71,72^, perceptual uncertainty^73,74^, and computational uncertainty induced by cognitive load^75^, all of which could influence exploration behavior in different ways. Thus, we expect future studies to increasingly focus on disentangling different sources of uncertainty and their effects on the exploration-exploitation dilemma.

Additionally, while our four-armed bandit task was designed to provide a richer choice set beyond two options, magnifying the difference between directed and random exploration, it still pales in comparison to the complexity of many real world problems. Since participants may be more likely to engage in directed exploration in highly complex or highly structured domains^21,22,70^, an important future direction will be to understand how environmental structure modulates changes in learning as a function of cognitive limitations.

Lastly, we have also only looked at multi-armed bandits in which participants only gain positive rewards or earn nothing when exceeding the time limit. We did not, however, probe how exploration behavior changes in the domain of losses^76,77^ or risky outcomes^33,78^. Since the distribution of rewards can affect participants’ learning^79^ and losses have been shown to produce risk-seeking under time pressure^45,46^, studying this domain will be a crucial next step.

### Conclusions

We studied the interplay of human exploration strategies and cognitive limitations imposed by time pressure, showing that participants are sensitive to the costs and benefits of different computations. Put under time pressure, people were less influenced by uncertainty, less value-directed, and repeated past choices more often. These behavioral changes are linked to the cognitive costs of reasoning about rewards and uncertainty. Exploitative choices (i.e., high reward expectations) were generally faster, while exploratory choices (i.e, high relative or total uncertainty) were slower. Taken together, our results suggest that people display a resource-rational sensitivity to the cost-benefits of different exploration strategies under externally imposed limitations on cognitive resources.

## Methods

### Participants and design

We recruited 99 participants (36 female, aged between 21 and 69 years; *M* = 34.82; *SD* = 10.1) on Amazon Mechanical Turk (requiring 95% approval rate and 100 previously approved HITs). Participants were paid $3.00 for taking part in the experiment and a performance contingent bonus of up to $4.00 (calculated based on the performance of one randomly selected round). Participants spent 13.0 ± 5.6 min on the task and earned $5.87 ± $0.91 in total. The study was approved by the Ethics Committee of the Max Planck Institute for Human Development and all methods were carried out in accordance with relevant guidelines and regulations.. Informed consent was obtained from all subjects.

We used a $2 \times 4$ within-subject design to examine how the presence or absence of time pressure and the payoff structure of the task (see Fig. 1c and Table 1) influenced choices and reaction times. In total, the experiment consisted of 40 rounds with 20 trials each. In each round, a condition was sampled (without replacement) from a pre-randomized list, such that each combination of time pressure and payoff structure was repeated five times, with a total of 100 trials in each.

### Materials and procedure

Participants were required to complete three comprehension questions and two practice rounds (one with unlimited time and one with limited time) consisting of 5 trials each before starting the experiment. Each of the 40 rounds was presented as a four-armed bandit task, where the four options were randomly mapped to the [*Q*, *W*, *O*, *P*] keys on the keyboard (Fig. 1a). Selecting an option by pressing the corresponding key yielded a reward sampled from a normal distribution, where the mean and variance was defined by the round’s payoff structure (Fig. 1c and Table 1). Participants completed 20 trials in each round and were told to acquire as many points as possible.

Before starting a round, participants were informed whether it was an unlimited or a limited time round. In unlimited time rounds, participants could spend as much time as they needed to reach a decision, upon which they were given feedback about the obtained reward (displayed for 400 ms) before continuing to the next trial (Fig. 1b). In limited time rounds, participants were instructed to decide as fast as possible. If a decision took longer than 400 ms, they forfeited the reward they would have earned (presented to them as a crossed-out number with an additional sad smiley; Fig. 1b). We used the same feedback period of 400 ms to display feedback about obtained rewards in both limited and unlimited time rounds.

We applied a random shifting of rewards across rounds (i.e., different minimum and maximum reward) to prevent participants from immediately recognizing when they had chosen the optimal option. For each round, we sampled a value from a uniform distribution $\mathcal {U}(30,60)$, which was then added to the rewards. Together with random shifting, we also truncated rewards such that they were always larger than zero. In order to convey intuitions about the random shift of rewards, payoffs were presented using a different fictional currency in each round (e.g., ß, Þ, $\vartheta$), such that the absolute value was unknown, but higher were always better.

At the end of each round, participants were given feedback about their performance in terms of the bonus they would gain (in USD) if this was the round selected for determining the bonus. The bonus was calculated as a percentage of the total possible performance, raised to the power of 4 to accentuate differences in the upper range of performance: $\text{Bonus} = \left( \frac{\text{total} \; \text{reward} \;\text{gained} }{\text{mean} \; \text{reward} \; \text{of} \; \text{best} \; \text{option} \times 20 trials }\right) ^4 \times \$4.00$

### Payoff conditions

We used four different payoff conditions as a within-participant manipulation (Table 1 and Fig. 1c). Each payoff condition specified the mean $\mu _j$ and variance $\sigma ^2_j$ of the reward distribution $R_j \sim \mathcal {N}(\mu _j,\sigma ^2_j)$ for each option *j*. Each distribution was randomly mapped to one of the four [*Q*, *W*, *O*, *P*] keys of the keyboard in each round. The Iowa Gambling Task (IGT) is a classic design that has been related to a variety of clinical and neurological factors affecting decision-making^54,80^. We implemented a reward condition inspired by the IGT such that there are two high and two low reward options, with a low and high variance version of each. We also constructed two conditions with equally spaced means, but with either uniformly low variance or uniformly high variance. Lastly, the equal means condition had identical means and gradually increasing variance, such that we can observe the influence of uncertainty independent of mean reward.

### Model-based analyses

### Bayesian mean tracker

The Bayesian mean tracker (BMT) learns a posterior distribution over the mean reward $\mu _j$ for each option *j*. Rewards are assumed to be normally distributed with a known variance but unknown mean. The prior distribution of the mean is also a normal distribution. This implies that the posterior distribution for each mean is also a normal distribution:

3 $$\begin{aligned} p_t(\mu _{j}|\mathcal {D}_{t-1}) = \mathcal {N}(m_{j,t},v_{j,t}) \end{aligned}$$

where $p_t$ is the posterior distribution at trial *t* and $\mathcal {D}_{t-1}$ denotes the observed rewards and choices up to and including trial *t* (for all options). For a given option *j*, the posterior mean $m_{j,t}$ and variance $v_{j,t}$ at trial *t* are only updated when it has been selected at trial *t*:

4 $$\begin{aligned} m_{j,t}&= m_{j,t-1} + \delta _{j,t}G_{j,t}\left[ y_t-m_{j,t-1}\right] \end{aligned}$$

5 $$\begin{aligned} v_{j,t}&= \left[ 1 - \delta _{j,t}G_{j,t}\right] v_{j,t-1} \end{aligned}$$

where $\delta _{j,t}=1$ if option *j* is chosen on trial *t*, and 0 otherwise. Additionally, $y_t$ is the observed reward at trial *t*, and $G_{j,t}$ is defined as:

6 $$\begin{aligned} G_{j,t} = \frac{v_{j,t-1}}{v_{j,t-1}+ \theta _\epsilon ^2} \end{aligned}$$

where $\theta _\epsilon ^2$, referred to as the error variance, is the variance of the rewards around the mean.

Intuitively, the estimated mean of the chosen option $m_{j,t}$ is updated based on prediction error, which is the difference between the observed reward $y_t$ and the prior expectation $m_{j,t-1}$, multiplied by learning rate $G_{j,t} \in [0,1]$. At the same time, the estimated variance $v_{j,t}$ of the chosen option is reduced by a factor $1 - G_{j,t}$. The error variance ($\theta _\epsilon ^2$) can be interpreted as an inverse sensitivity, where smaller values result in more substantial updates to the mean $m_{j,t}$, and larger reductions of uncertainty $v_{j,t}$. We set the prior mean to $m_{j,0}=0$ based on the (unshifted) expectation across payoff conditions, and the prior variance is set to $v_{j,0}=55*20$, which is also the expectation across payoff conditions, scaled by a constant multiple of 20. We use unshifted reward values (i.e., before adding the shift $\sim \mathcal {U}(30,60)$ were observed by participants), with the means in each condition centered on 0. For our model-based analysis, the error variance $\theta _\epsilon ^2$ was set to the true underlying variance of the chosen option.

### Hierarchical Bayesian regression models

### Mixed effects regressions

All Bayesian mixed effects regression models used Hamiltonian Markov chain Monte Carlo (MCMC) with a No-U-Turn sampler^81^ and were implemented using brms^82^. All models used generic, weakly informative priors $\sim \mathcal {N}(0,1)$ with the proposal acceptance probability set to .99. In all cases, participants were assigned a random intercept and all fixed effects also had corresponding random effects following recommendations to apply a maximal random-effects structure^83^. All models were estimated over four chains of 4000 iterations, with a burn-in period of 1000 samples.

### Softmax choice model

The softmax choice model was estimated hierarchically using custom code written in STAN. Formally, we assume that the $\alpha$- and $\beta$-coefficients (see Eq. 2) for each participant are drawn independently from a normal distribution:

7 $$\begin{aligned} \alpha _i^\text {limited}, \alpha _i^\text {unlimited}, \beta _i^\text {limited}, \beta _i^\text {unlimited}, \gamma _i^\text {limited}, \gamma _i^\text {unlimited} \sim \mathcal {N}(\mu _0, \sigma _0^2). \end{aligned}$$

For simplicity, we use $\alpha _i$, $\beta _i$, and $\gamma _i$ in Eq. (2) to refer to $\theta _i = \theta _i^\text {limited} + \mathbbm {1}\theta _i^\text {unlimited}$, where $\theta \in [\alpha , \beta , \gamma ]$ and $\mathbbm {1}=1$ for unlimited time rounds, and 0 otherwise. We used Hamiltonian MCMC with a No-U-Turn sampler^81^ to estimate the group-level mean $\mu _0$ and variance over participants $\sigma _0^2$ for $\alpha$, $\beta$, and $\gamma$, and their interaction with time pressure. We used the following priors on the group-level parameters:

8 $$\begin{aligned} \mu _0&\sim \mathcal {N}(0, 1) \end{aligned}$$

9 $$\begin{aligned} \sigma _0^2&\sim \mathcal {N}(0, 1) \in (0, \infty ) \end{aligned}$$

The posterior mean and uncertainty estimates of the BMT were standardized between [0,1] before being entered into the regression. The model was estimated over four chains of 4000 iterations, with a burn-in period of 1000 samples, and with the proposal acceptance probability set to 0.99.

### RTs

The RT regression used the same Bayesian mixed effects framework as above, with log-transformed RTs as the dependent variable. 1 ms was added to each RT to avoid $\log (0)$, with the raw RTs truncated at a maximum of 5000 ms. Both dependent and independent variables were standardized to a mean of 0 and unit variance.

### LBA

Formally, the LBA assumes that, after an initial period of non-decision time $\tau$, evidence for option *j* accumulates linearly at a rate of $v_{j}$, starting from an initial evidence level $p_{j} \sim \mathcal {U}(0,A)$. Evidence accumulates for each option *j* until a threshold $b = A + k$ is reached. We follow the Bayesian implementation proposed by Ref.^61^ and assume that the priors for the drift rates stem from truncated normal distributions

10 $$\begin{aligned} v_{j} \sim \mathcal {N}(2,1) \in (0, \infty ). \end{aligned}$$

Additionally, we assume a uniform prior on non-decision time

11 $$\begin{aligned} \tau \sim \mathcal {U}(0,1), \end{aligned}$$

and a truncated normal prior on the maximum starting evidence

12 $$\begin{aligned} A \sim \mathcal {N}(0.5,1) \in (0, \infty ). \end{aligned}$$

Finally, we reparameterized the model by shifting *b* by *k* units away from *A*, and put a truncated normal distribution as the prior on the resulting relative threshold *k*:

13 $$\begin{aligned} k \sim \mathcal {N}(0.5,1) \in (0, \infty ). \end{aligned}$$

We estimated the LBA parameters (see Fig. S12) for each participant in every round separately using No-U-Turn Hamiltonian MCMC^81^, with reaction times truncated at 5000 ms. The drift rate regression used the same Bayesian mixed effects framework as above, with both DVs and IVs standardized to a mean of 0 and unit variance.

## Supplementary Information

Supplementary Information.

## Data Availability

Code and data are publicly available at https://osf.io/v4dua/.
