# Supplementary material for: Time pressure changes how people explore and respond to uncertainty
Source: Sci Rep. 2022 Mar 8;12:4122. doi: 10.1038/s41598-022-07901-1 (PMC8904509; doi:10.1038/s41598-022-07901-1)
Supplement: Supplementary file 1 — Supplementary Information. [file 41598_2022_7901_MOESM1_ESM.pdf]

Supporting information

Time pressure changes how people explore and respond to uncertainty

Charley M. Wu, Eric Schulz, Timothy J. Pleskac, & Maarten Speekenbrink

Statistics

Comparisons.

Both frequentist and Bayesian statistics are reported throughout this paper. Frequentist tests are reported as Student's t-tests (specified as either paired or independent). Each of these tests are accompanied by a Bayes factors (BF) to quantify the relative evidence the data provide in favor of the alternative hypothesis (H_A) over the null (H_0). This is done using the default two-sided Bayesian t-test for either independent or dependent samples, where both use a Jeffreys-Zellner-Siow prior with its scale set to $\sqrt{2}/2$, as suggested by Ref[1]. All statistical tests are non-directional as defined by a symmetric prior.

Correlations.

For testing linear correlations with Pearson's r, the Bayesian test is based on Jeffreys[2] test for linear correlation and assumes a shifted, scaled beta prior distribution $B(\frac{1}{k},\frac{1}{k})$ for r, where the scale parameter is set to $k=\frac{1}{3}$[3]. Note that when performing group comparisons of correlations computed at the individual level, we report the mean correlation and the statistics of a single-sample t-test comparing the distribution of z-transformed correlation coefficients to $\mu=0$.

For testing rank correlations with Kendall's tau, the Bayesian test is based on parametric yoking to define a prior over the test statistic[4], and performing Bayesian inference to arrive at a posterior distribution for r_τ. The Savage-Dickey density ratio test is used to produce an interpretable Bayes Factor.

ANOVA.

We use a two-way analysis of variance (ANOVA) to compare the means of $p \geq 2$ samples based on the F distribution. In general terms, we can define ANOVA as a linear model:

$$\mathbf{y} = \mu\mathbf{1} + \sigma\mathbf{X}\boldsymbol{\theta} + \boldsymbol{\varepsilon} \tag{1}$$

where $\mathbf{y}$ is a vector of N observations, μ is the aggregate mean, $\mathbf{1}$ is a column vector of length N, σ is the scale factor, $\mathbf{X}$ is the $N \times p$ design matrix, $\boldsymbol{\theta}$ is a column vector of the standardized effect sizes, and $\boldsymbol{\varepsilon}$ is a column vector containing the i.i.d. errors where $\varepsilon_i \overset{\text{i.i.d}}{\sim} \mathcal{N}(0,\sigma^2)$.

We assume independent g-priors[5] for each effect size $\theta_1 \sim \mathcal{N}(0,g_1\sigma^2),\cdots,\theta_p \sim \mathcal{N}(0,g_p\sigma^2)$, where each g-value is drawn from an inverse chi-square prior with a single degree of freedom $g_i \overset{\text{i.i.d}}{\sim}$ inverse-$\chi^2(1)$. For μ and σ^2 we assume a Jeffreys[6] prior. Following Ref[7], we compute the Bayes factor by integrating the likelihoods with respect to the prior on parameters, where Monte Carlo sampling was used to approximate the g-priors. The Bayes factor reported in the text can be interpreted as the log-odds of the model relative to an intercept-only null model.

Supplementary Behavioral results

Raw RTs.

Figure S3a shows the distribution of participant reaction times (RTs) split by time pressure and payoff conditions. Using a two-way within subject ANOVA, we found that participants (unsurprisingly) responded faster in limited time ($F(1,98)=13.8$, $p<.001$, $\eta^2=.016$, $BF>100$), but with no differences across payoff conditions ($F(3,98)=0.684$, $p=.562$, $\eta^2=.002$, $BF=0.005$). Additionally, we find that participants sped up over trials (average correlation: $\bar{r}=-.54$; one-sample t-test against zero using

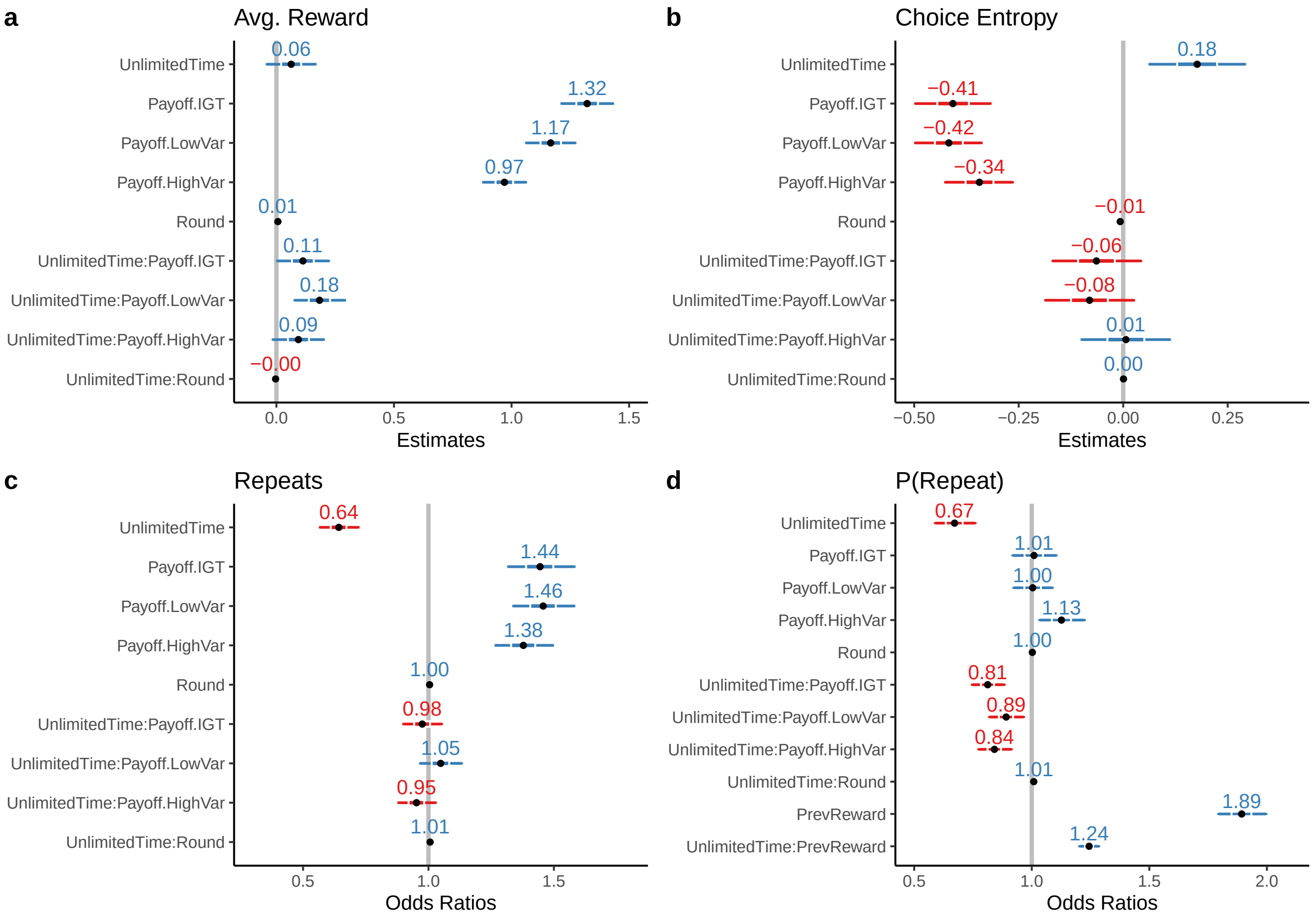

Figure S1. Regression coefficients. Visualization of regression coefficients, corresponding to the models in Table S1. The vertical grey line represents chance, and estimates above are in blue, while estimates below are in red (irrespective of significance). The inner horizontal line indicates the 50% HDI and the outer line indicates 89% HDI.

z-transformed correlation coefficients: $t(98) = -17.6$, $p < .001$, $d = 1.8$, $BF > 100$; Fig. S3b), with a strong speed up in unlimited time (paired t-test comparing z-transformed correlation coefficients: $t(98) = 4.5$, $p < .001$, $d = 0.5$, $BF > 100$). We see a similar speed-up over rounds (average correlation: $t(98) = -9.4$, $p < .001$, $d = 0.9$, $BF > 100$; Fig. S3c), which was also more pronounced under unlimited time ($t(98) = 4.4$, $p < .001$, $d = 0.5$, $BF > 100$).

Too slow analyses

We also analysed the pattern of "too slow" responses that exceeded 400ms during the limited time condition, where participants received no reward. Using a one-way within subject ANOVA, we found no differences in the number of "too slow" responses across payoff conditions ($F(3,98) = 0.088$, $p = .966$, $\eta^2 = .001$, $BF = 0.012$; Fig. S4a). Too slow responses tended to follow lower-valued reward observations (paired t-test: $t(93) = -6.2$, $p < .001$, $d = 0.8$, $BF > 100$; omitting 5 participants who never had slow responses; Fig. S4b). However, this effect is mediated by trial number, since the vast majority of "too slow" responses occurred on the second trial (Fig. S4c). Since the learning curves in Fig. **??**a indicate gradual increases in reward over trials (except for the Equal Means condition), a tendency for "too slow" responses to take place during early trials will generally correspond to lower reward observations.

In order to simultaneously model the influence of payoff conditions, trials, and reward observations on the tendency to produce a "too slow" response, we fit a Bayesian logistic mixed-effects model (Fig. S4d).

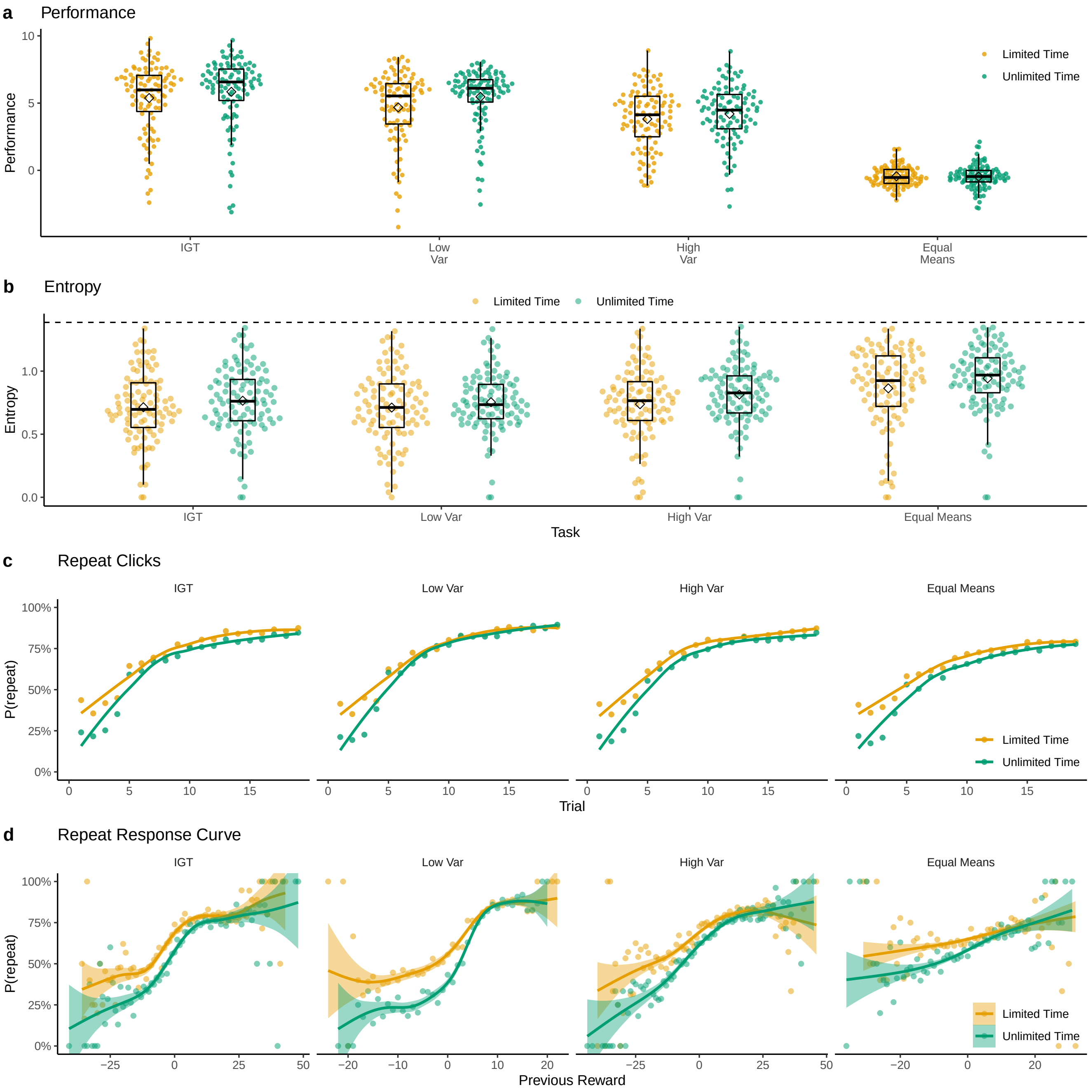

Figure S2. Raw Behavioral Data Split By Conditions. a) Average performance across payoff and time conditions. Each dot is a single participant, with overlaid Tukey boxplots and diamonds indicating the group means. **b**) Entropy of choices by payoff and time conditions, where the dashed line indicates a random baseline. **c**) Repeat clicks as a function of trial, where each dot is the group mean and the lines are a locally smoothed regression. **d**) Repeat clicks as a function of previous reward value. Ribbons indicate the 95% CI.

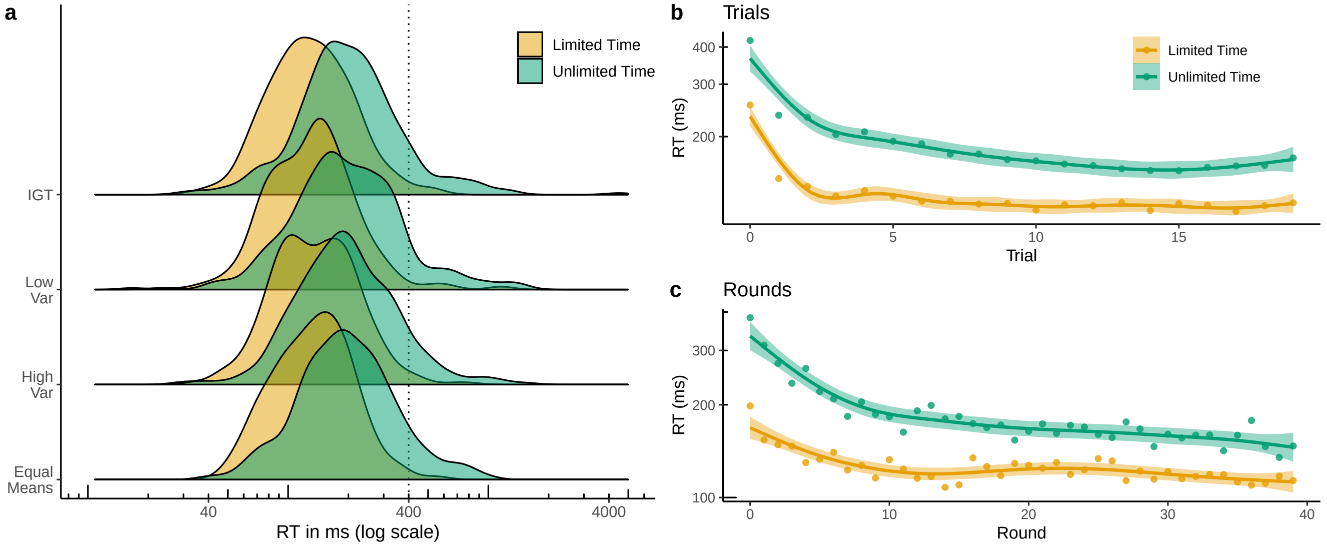

Figure S3. Reaction Times (RTs). All RTs are shown in milliseconds (ms) and on a log scale. Outliers greater than 5000ms are omitted from the plots, but not from the analyses. **a**) RT distributions separated by payoff and time conditions. The dashed line indicates the 400 ms limit for limited time choices. **b**) RTs as a function of trial. Each dot is the aggregate mean, with the lines and ribbons indicating the mean and 95% confidence intervals of a generalized additive regression. **c**) RTs as a function of Round.

We again found no influence of payoff condition (all estimates overlapping with an odds ratio of 1). However, we find a strong effect of trial (OR: 0.88 $[.86, .91]$) and a reliable but small effect of previous reward (OR: 0.99 $[.98, .99]$), such that both later trials and larger rewards observations were less likely to produce “too slow” responses.

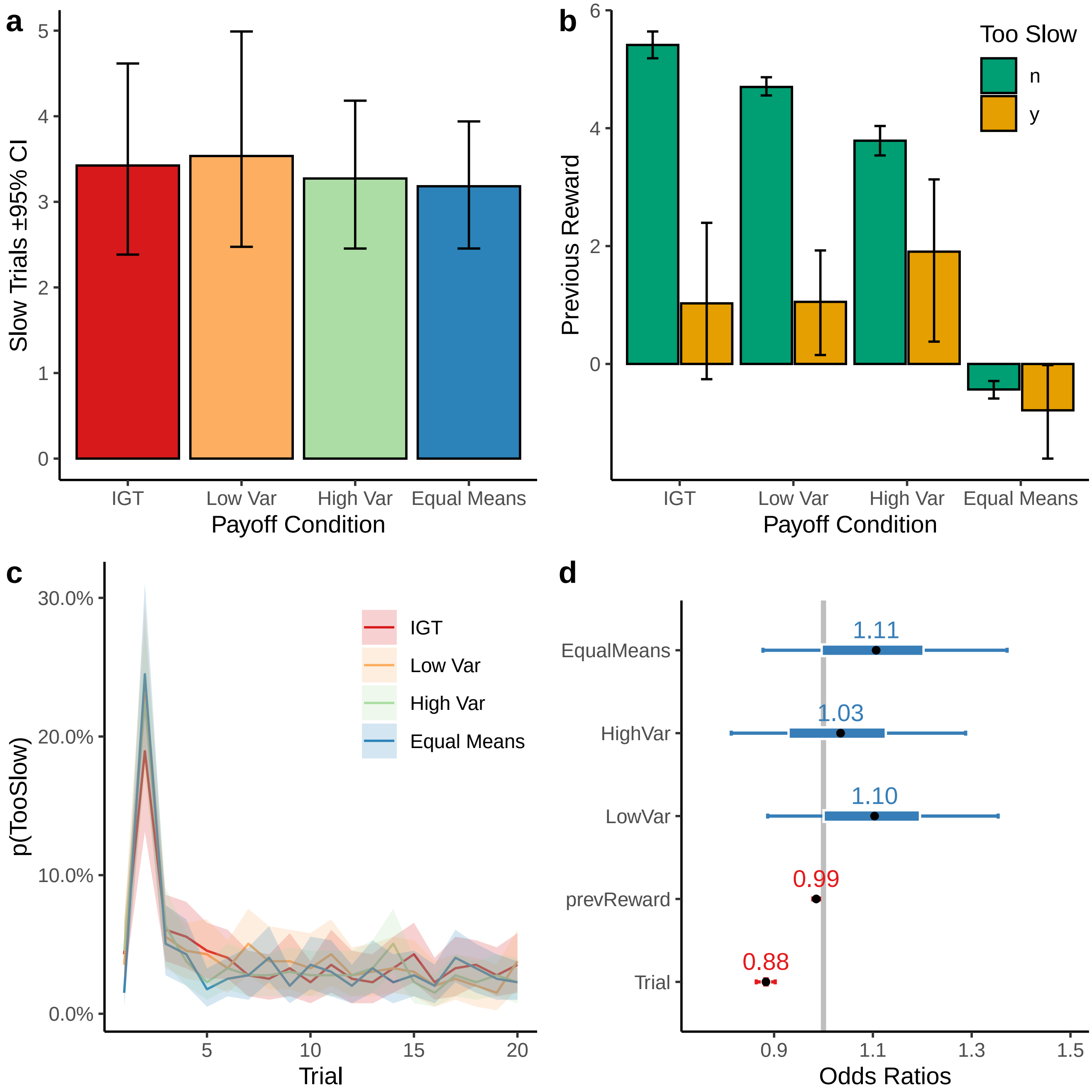

Figure S4. **"Too slow" analyses**. All analyses were performed solely on limited time rounds. **a**) The average number of slow trials (>400ms), separated by payoff condition. Bars indicate group means, while error bars show the 95% CI. **b**) Average reward observation immediately preceding a trial that was either too slow (orange) or not (green), separated by Payoff condition. **c**) The probability of a response being too slow as a function of trial, with each line showing the group means for each payoff condition and the ribbons indicating the 95% CI. **d**) Regression coefficients of the Bayesian logistic mixed effects model, depicted as odds ratios with the vertical grey line indicating chance. The inner horizontal line indicates the 50% HDI and the outer line indicates the 89% HDI.

Table S1. Bayesian Mixed Effects Regression: Experimental Manipulations

	Avg. Reward *Estimate*	Choice Entropy *Estimate*	Repeats *Odds Ratio*	P(Repeat) *Odds Ratio*
Intercept	-1.04	0.36	1.94	2.73
	[-1.13, -0.95]	[0.19, 0.52]	[1.55, 2.45]	[2.13, 3.47]
UnlimitedTime	0.06	0.18	0.64	0.67
	[-0.06, 0.19]	[0.04, 0.32]	[0.55, 0.74]	[0.57, 0.78]
Payoff.IGT	1.32	-0.41	1.44	1.01
	[1.19, 1.46]	[-0.52, -0.30]	[1.29, 1.61]	[0.90, 1.13]
Payoff.LowVar	1.17	-0.42	1.46	1.00
	[1.04, 1.29]	[-0.52, -0.32]	[1.31, 1.61]	[0.91, 1.11]
Payoff.HighVar	0.97	-0.34	1.38	1.12
	[0.86, 1.08]	[-0.44, -0.25]	[1.25, 1.52]	[1.01, 1.25]
Round	0.01	-0.01	1.00	1.00
	[0.00, 0.01]	[-0.01, -0.00]	[1.00, 1.01]	[1.00, 1.01]
UnlimitedTime:Payoff.IGT	0.11	-0.06	0.97	0.81
	[-0.2, 0.25]	[-0.19, 0.07]	[0.88, 1.07]	[0.73, 0.90]
UnlimitedTime:Payoff.LowVar	0.18	-0.08	1.05	0.89
	[0.05, 0.32]	[-0.21, 0.05]	[0.95, 1.15]	[0.81, 0.98]
UnlimitedTime:Payoff.HighVar	0.09	0.01	0.95	0.84
	[-0.04, 0.22]	[-0.12, 0.14]	[0.87, 1.04]	[0.76, 0.93]
UnlimitedTime:Round	-0.00	0.00	1.01	1.01
	[-0.01, 0.00]	[-0.00, 0.00]	[1.00, 1.01]	[1.01, 1.01]
PrevReward				1.89
				[1.77, 2.02]
UnlimitedTime:PrevReward				1.24
				[1.19, 1.29]
**Random Effects**				
σ^2	0.13	0.41	12.69	0.01
τ_{00}	0.88	0.59	4.47	0.21
ICC	0.13	0.41	0.72	0.04
$N_{Participant}$	99	99	99	99
Observations	3960	3960	3960	76240
Bayesian R^2	0.43	0.46	0.65	0.26

Note: Each model was defined as `DV` $\sim$ `TimePressure * PayoffConditions * Round + (1 + TimePressure + PayoffConditions + Round | Participant)`, where `DV` is the dependent variable (columns), and `PayoffConditions` were defined using dummy coding, with the baseline being the Equal Means condition. We report the posterior mean and 95% highest posterior density (HPD) interval below in brackets. The 'Repeats' model is a Binomial regression based on 19 successive Bernoulli trials (since the first trial cannot be a repeat). The 'P(Repeat)' model is a logistic regression, with the previous reward value added as an additional predictor. Both the 'Repeats' and 'P(Repeat)' models are reported as Odds Ratios. σ^2 indicates the individual-level variance, τ_{00} indicates the variation between individual intercepts and the average intercept, and ICC is the intraclass correlation coefficient. Model coefficients are visualized in Figure S1.

Table S2. Bayesian Mixed Effects Logistic Regression: Choice Probability for Highest Variance Option

	IGT	Equal Means
	Odds Ratio	*Odds Ratio*
Intercept	0.91	0.24
	[0.69, 1.20]	[0.18, 0.33]
UnlimitedTime	1.11	1.45
	[0.80, 1.53]	[1.11, 1.87]
Round	0.83	1.00
	[0.68, 1.02]	[0.99, 1.02]
UnlimitedTime:Round	1.39	0.99
	[1.23, 1.57]	[0.98, 0.99]
**Random Effects**		
σ^2	0.00	0.02
τ_{00}	0.25	0.17
ICC	0.00	0.00
$N_{Participant}$	99	99
Observations	10230	19800
Bayesian R^2	0.194	0.134

Note: Each model was defined as `DV ~ TimePressure * Round + (1 + TimePressure + Round|Participant)`, where `DV` is the dependent dependent binary variable representing whether the highest variance option was chosen. In the IGT regression, we only consider choices where the two highest mean reward options were chosen ('O' and 'P'), where $DV = 1$ when 'P' was chosen, and zero otherwise. For the Equal Means condition, we include all choices. We report the posterior odds ratio and 95% highest posterior density (HPD) interval below in brackets. σ^2 indicates the individual-level variance, τ_{00} indicates the variation between individual intercepts and the average intercept, and ICC is the intraclass correlation coefficient.

Supplementary Model Results

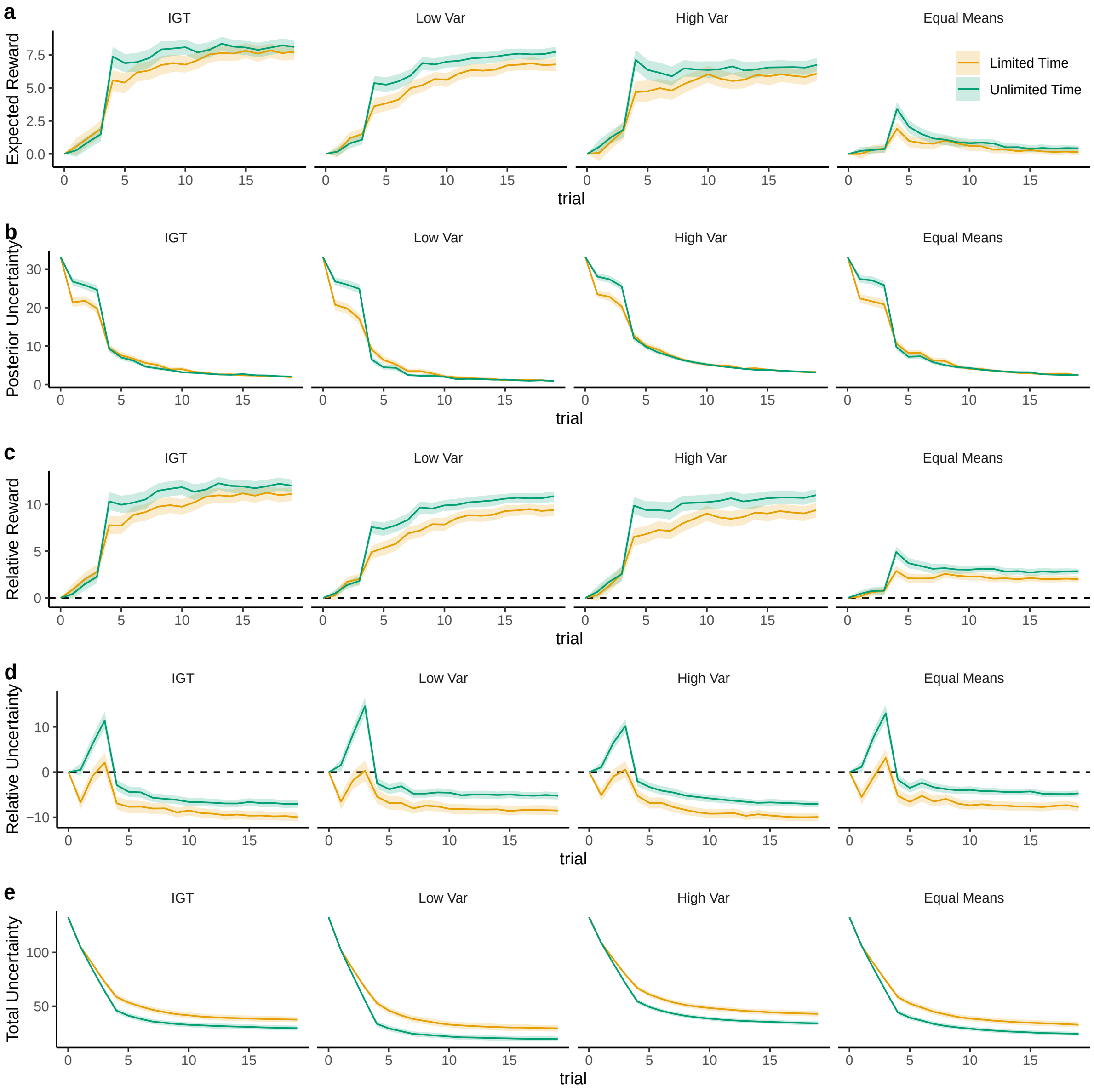

Figure S5. BMT predictions about the chosen option simulated for all participants. Lines indicate group means, with ribbons showing the 95% CI. **a**) Expected rewards (posterior mean) increase over successive trials, showing how the model tracks learning. The uptick in the Equal Means condition, followed by a decay back to zero indicates participants persevered after high reward observations stemming from the underlying variance, which then regressed back to the mean of 0. **b**) Posterior uncertainty (stdev) decays as participants exploit options with diminishing uncertainty. **c**) Relative reward shows the difference between the posterior mean of the chosen option and the average posterior mean of the unchosen options. Relative reward is always valued positively (dashed line indicates 0). **d**) Relative uncertainty shows the difference between the posterior uncertainty (stdev) of the chosen option and the average posterior uncertainty of the unchoen options. The early upticks indicates uncertainty directed exploration (substantially less in limited time), followed by exploitation as this value decays below zero (dashed line). **e**) Total uncertainty (stdev) decays monotonically, with a faster decline in unlimited time due to more uncertainty directed exploration.

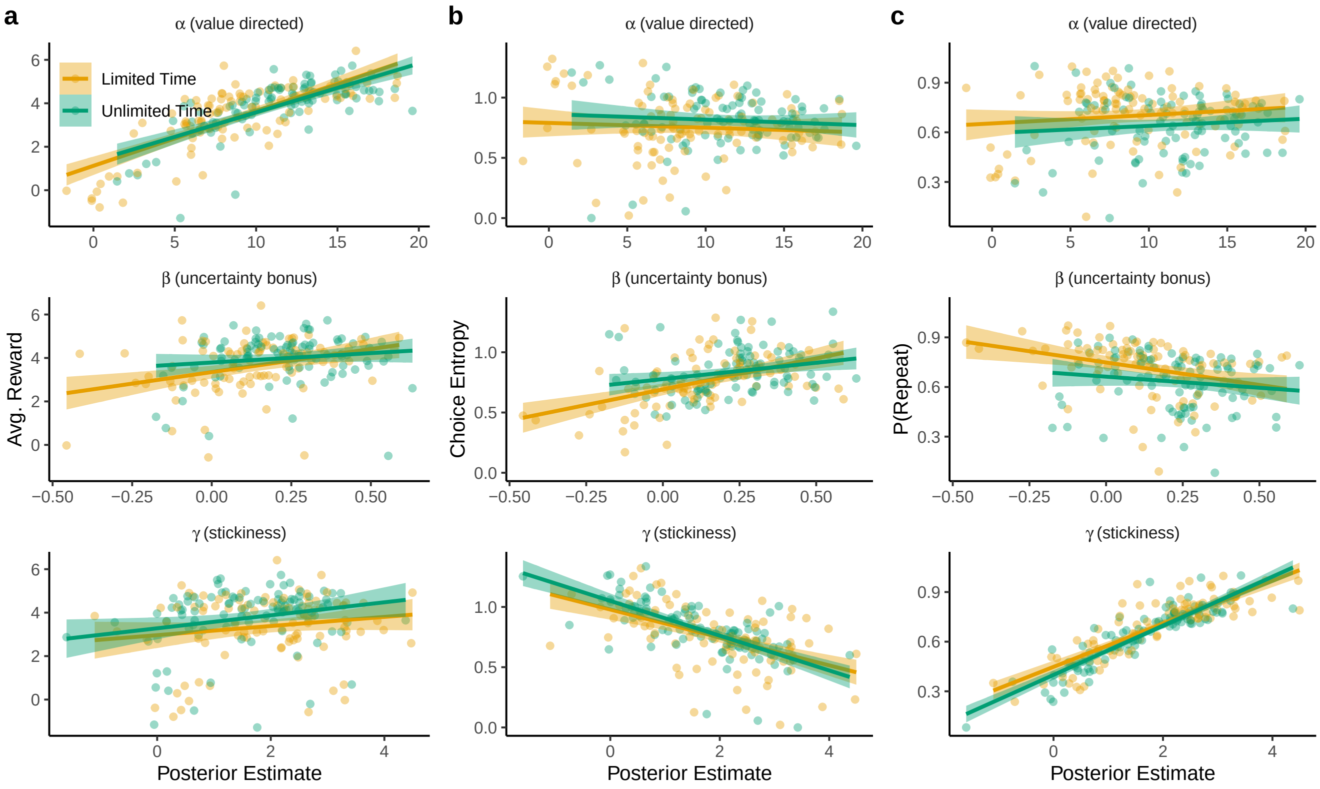

Figure S6. Comparison of Softmax parameters and behavior. Each dot shows the mean posterior for each participant in each time condition, while the lines and ribbons are a linear regression and 95% CI. **a**) Higher α estimates correspond to higher rewards in both conditions (unlimited time: $r_\tau = .55$, $p < .001$, $BF > 100$; limited time: $r_\tau = .55$, $p < .001$, $BF > 100$). However, we only find a reliable effect of β under time pressure ($r_\tau = .25$, $p < .001$, $BF > 100$), but not with unlimited time ($r_\tau = .13$, $p = .055$, $BF = .81$). This suggests that the lower overall performance under time pressure, may have a result of the reduction in uncertainty directed exploration (Fig. **??**a). We find no relationship between stickiness and rewards (unlimited time: $r_\tau = .13$, $p = .052$, $BF = .85$; limited time: $r_\tau = .08$, $p = .265$, $BF = .24$). **b**) We find no correlation between α and choice entropy (unlimited time: $r_\tau = -.13$, $p = .053$, $BF = .84$; limited time: $r_\tau = -.01$, $p = .835$, $BF = .13$). However, higher β estimates generated higher entropy choices in both conditions (unlimited time: $r_\tau = .26$, $p < .001$, $BF > 100$; limited time: $r_\tau = .36$, $p < .001$, $BF > 100$), while higher γ were related to lower entropy (unlimited time: $r_\tau = -.53$, $p < .001$, $BF > 100$; limited time: $r_\tau = -.41$, $p < .001$, $BF > 100$). **c**) Similar to choice entropy, we find no relationship between α and the frequency of repeat choices (unlimited time: $r_\tau = .06$, $p = .384$, $BF = .19$; limited time: $r_\tau = -.04$, $p = .545$, $BF = .16$). However, higher β estimates were correlated with less repeat choices in limited time ($r_\tau = -.30$, $p < .001$, $BF > 100$), and more weakly correlated in unlimited time ($r_\tau = -.19$, $p = .006$, $BF = 5.4$). Stickiness γ was unsurprisingly correlated with more repeat choices in both conditions (unlimited time: $r_\tau = .73$, $p < .001$, $BF > 100$; limited time: $r_\tau = .65$, $p < .001$, $BF > 100$). In all plots, Tukey's fence has been applied to omit outliers for clearer visualizations, but all data are included in the statistical tests.

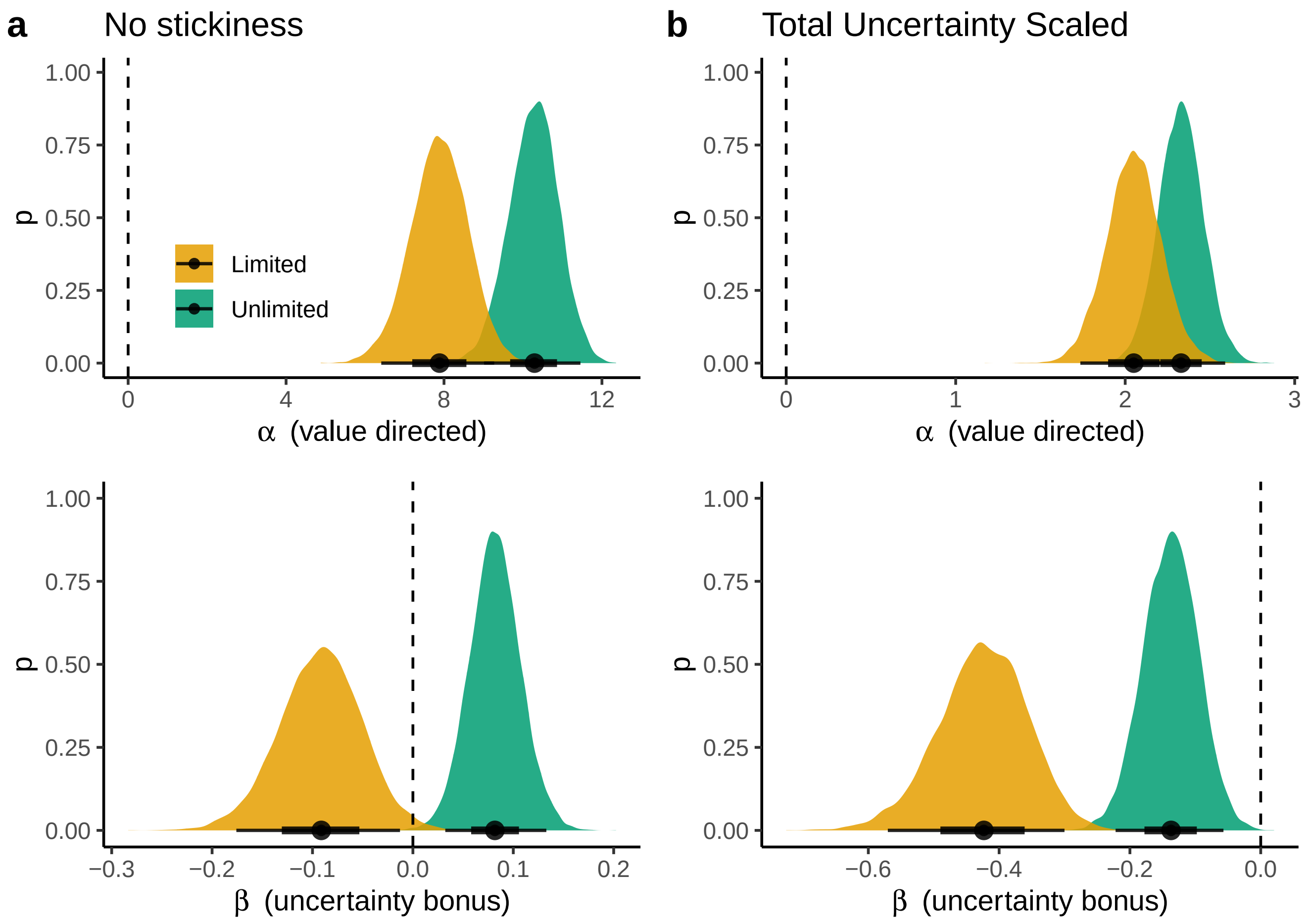

Figure S7. Alternative Softmax Model Posteriors. Posterior estimates for alternative formulations of the softmax model. **a**) Variant without stickiness, which yields negative uncertainty bonus β estimates for limited time. **b**) Variant, also without stickiness, but where the value-directed component was scaled by the total uncertainty (across options) as a method to regulate higher random exploration when the total uncertainty is high (following Ref[8]; see Fig. S8 for details). Here, we get negative uncertainty bonus β estimates for both conditions. Both models provide worse fits to the data (Fig. S8) compared to the sticky softmax model reported in Figure **??**a .

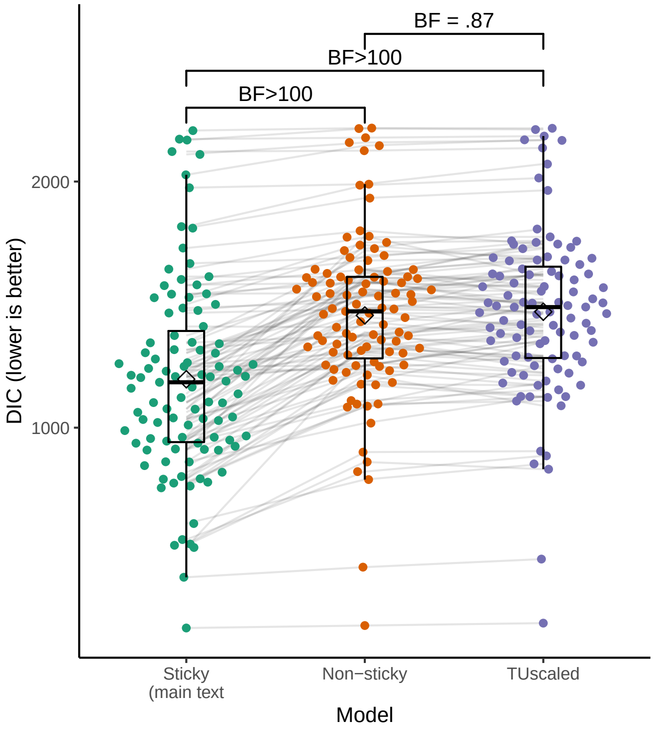

Figure S8. Model comparison. Comparing three variants of the hierarchical softmax choice model using Deviance Information Criterion[9] $DIC = -2\log \mathbb{E}_{\theta} p(y|\theta) + 2p_D$, where the effective number of parameters is defined as $p_D = \mathbb{V}_{\theta}\left(-2\log p\left(y|\theta\right)\right)$. The sticky model is reported in the main text, while the non-sticky model omits the γ term. Lastly, the total uncertainty scaled model (TUscaled) also omits the stickiness parameter, but rescales the value estimates going into the softmax function by the total uncertainty across all four options to account for changes in random exploration as a function of total uncertainty[8]: $Q_{j,t} = \frac{\alpha(m_{j,t}+\beta\sqrt{v_{j,t}})}{\sum_k \sqrt{v_{k,t}}}$. Each dot is a single participant (connected by lines across models),with overlaid Tukey boxplots and the diamond indicating the group mean. The significance tests are Bayes Factors (BF) corresponding to paired Bayesian t-tests. The sticky model beats the non-sticky model ($t(98) = -13.2$, $p < .001$, $d = 0.7$, $BF > 100$), the sticky model beats the TUscaled model ($t(98) = -15.6$, $p < .001$, $d = 0.7$, $BF > 100$), and there are no reliable differences between the non-sticky and TUscaled models ($t(98) = -2.1$, $p = .040$, $d = 0.0$, $BF = .87$).

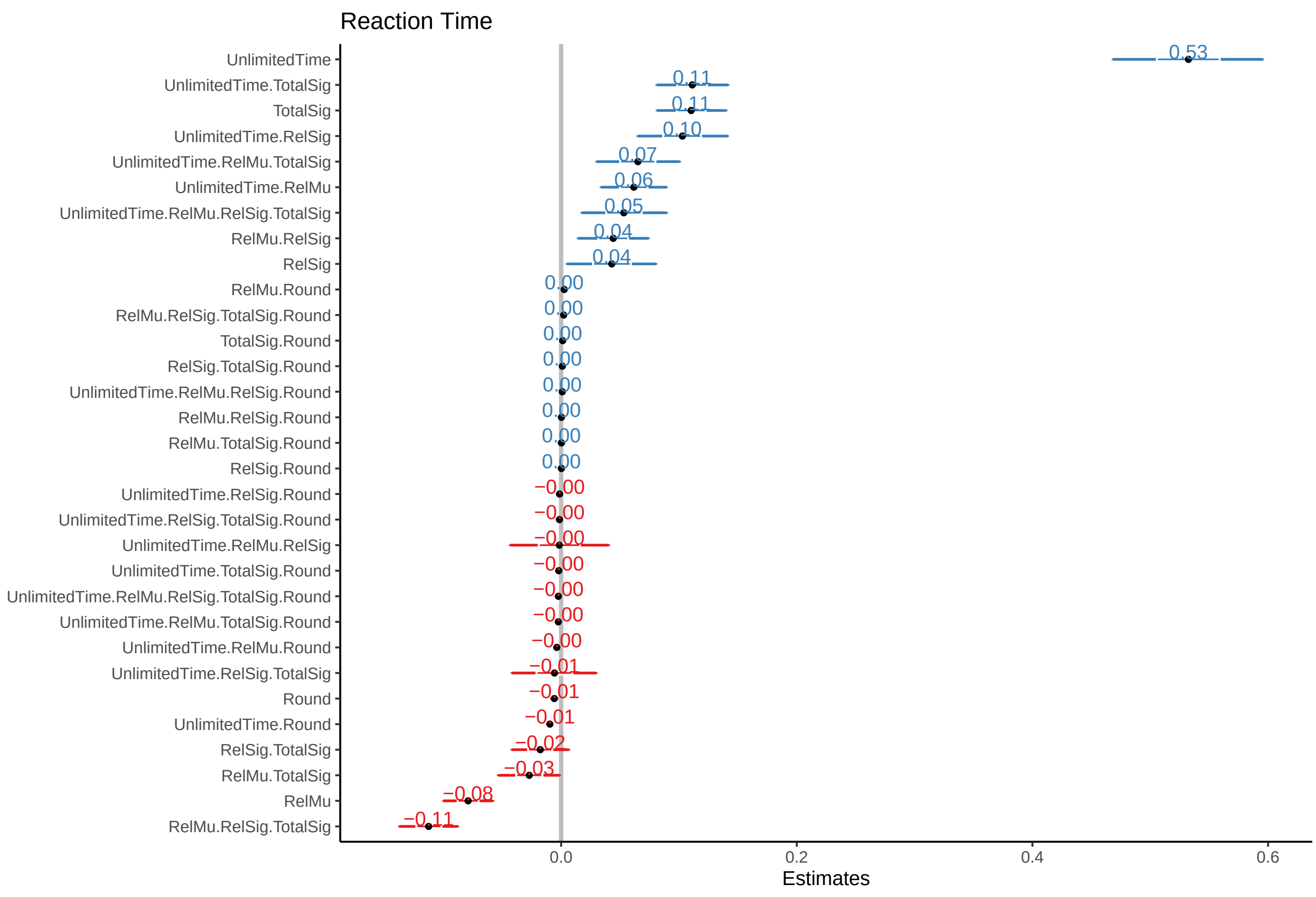

Figure S9. Coefficient plot of RT mixed effects regression. Posterior estimates of the Bayesian mixed effects regression predicting (log) RT. The mean posterior estimate is displayed numerically and indicated by the black dot, while the 95% HPD is illustrated by the length of the horizontal line. Coefficients are sorted by largest to smallest, with blue and red colors corresponding to estimates that are above or below 0, respectively, but do not indicate whether the difference is meaningful. See Figs. S10-S11 for interaction plots. RelMu: relative reward; RelSig: relative uncertainty; TotalSig: total uncertainty.

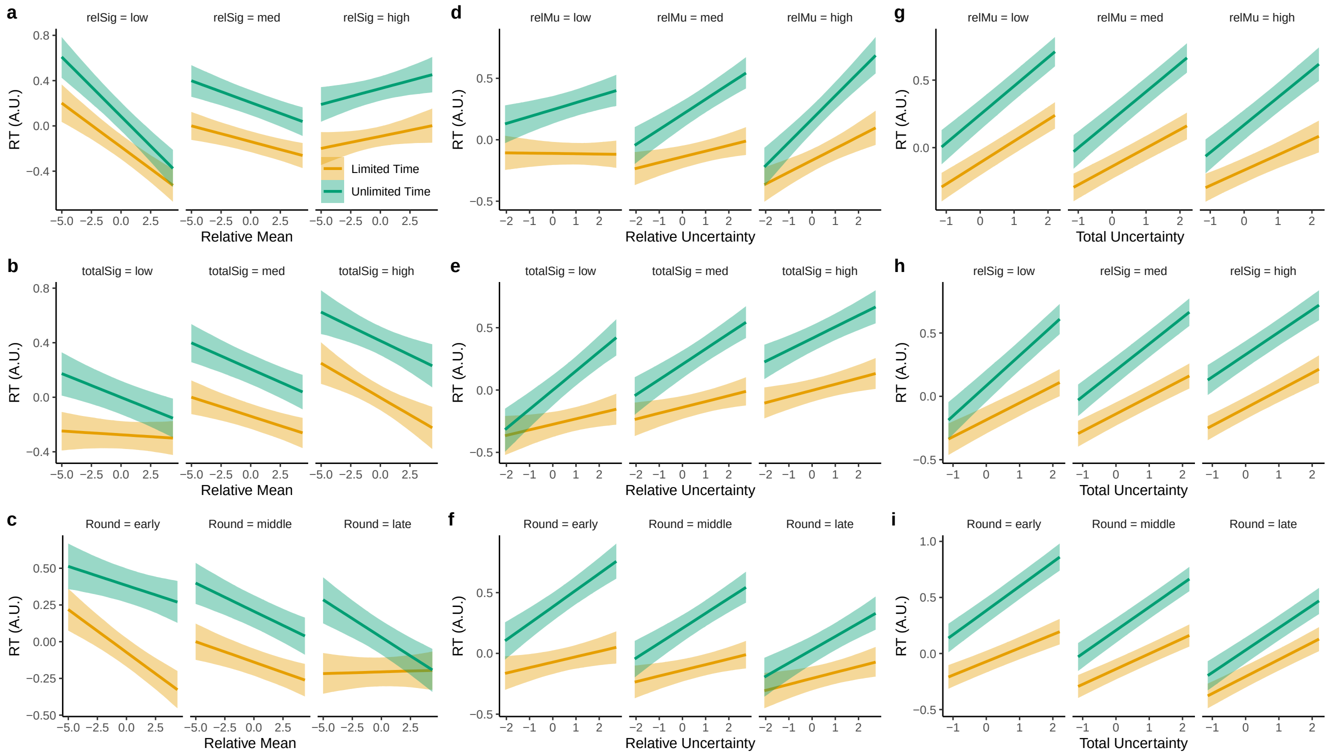

Figure S10. Marginal interaction plots for RT mixed effects regression. Marginal interactions of the RT Bayesian mixed effects regression illustrated in Fig. S9. Interactions are grouped in terms of relative means (**a-c**), relative uncertainty (**d-f**), and total uncertainty (**g-i**). Continuous variables are split into discrete $[low, med, high]$ levels, based on $[mean - sd,\ mean,\ mean + sd]$. RelMu: relative reward; RelSig: relative uncertainty; TotalSig: total uncertainty.

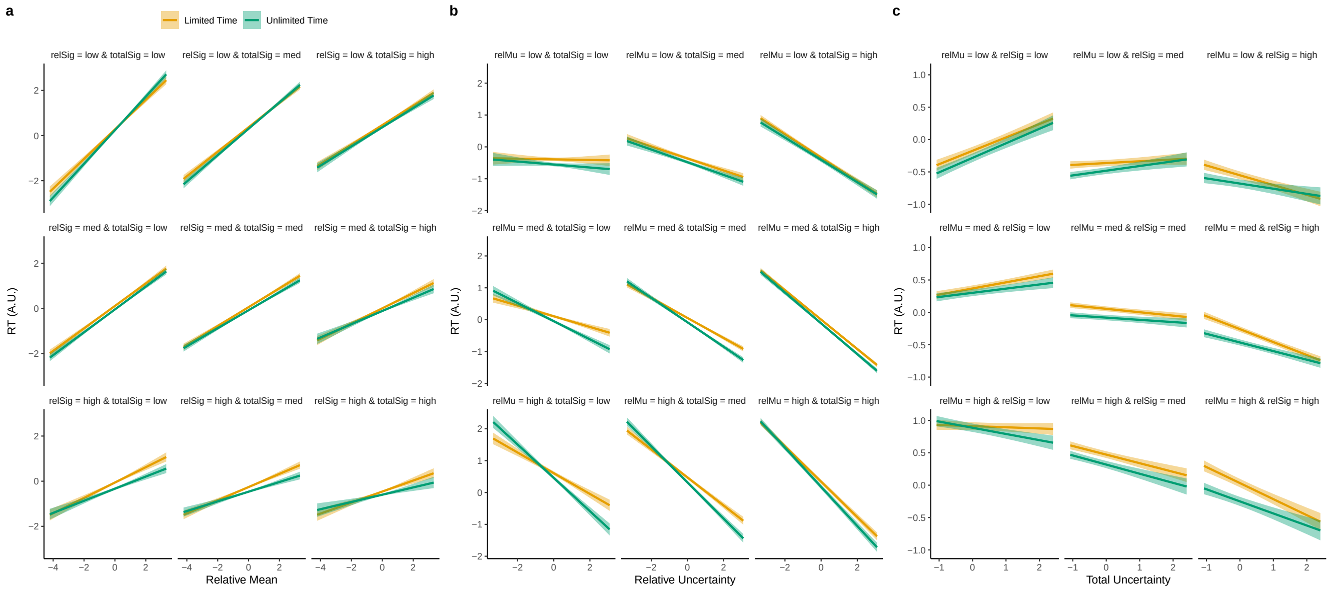

Figure S11. Four-way interactions for RT mixed effects regression. Four-way interactions of the RT Bayesian mixed effects regression illustrated in Fig. S9. Interactions are grouped in terms of relative means (**a**) and relative uncertainty (**b**). Continuous variables are split into discrete $[low, med, high]$ levels, based on $[mean - sd$, $mean$, $mean + sd]$, with relative uncertainty (relSig) increasing top to bottom (rows) and total uncertainty (totalSig) increasing from left ro right (columns). RelMu: relative reward; RelSig: relative uncertainty; TotalSig: total uncertainty.

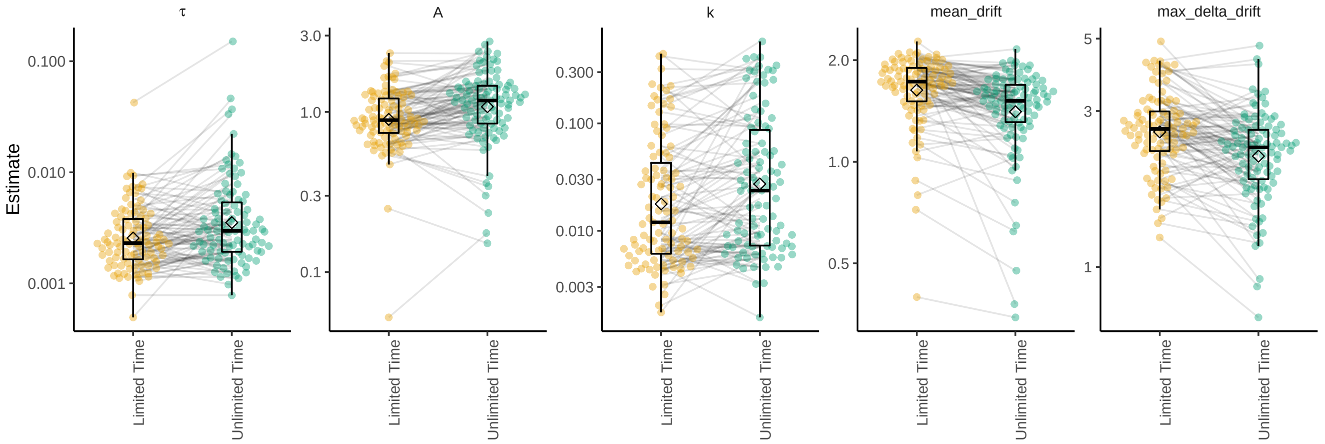

Figure S12. LBA parameters. Mean posterior parameter estimates of the LBA model, where each pair of connected dots is a single participant. Tukey boxplots show the group statistics, with the diamond indicating group means. τ is the non-decision time, A is the maximum starting evidence, k is the relative threshold, mean_drift is the average drift rate across all four options $\frac{1}{4}\sum_j v_j$, and max_drift_diff is the largest pairwise difference in drift rates $\max_{i \neq j} |v_i - v_j|$.

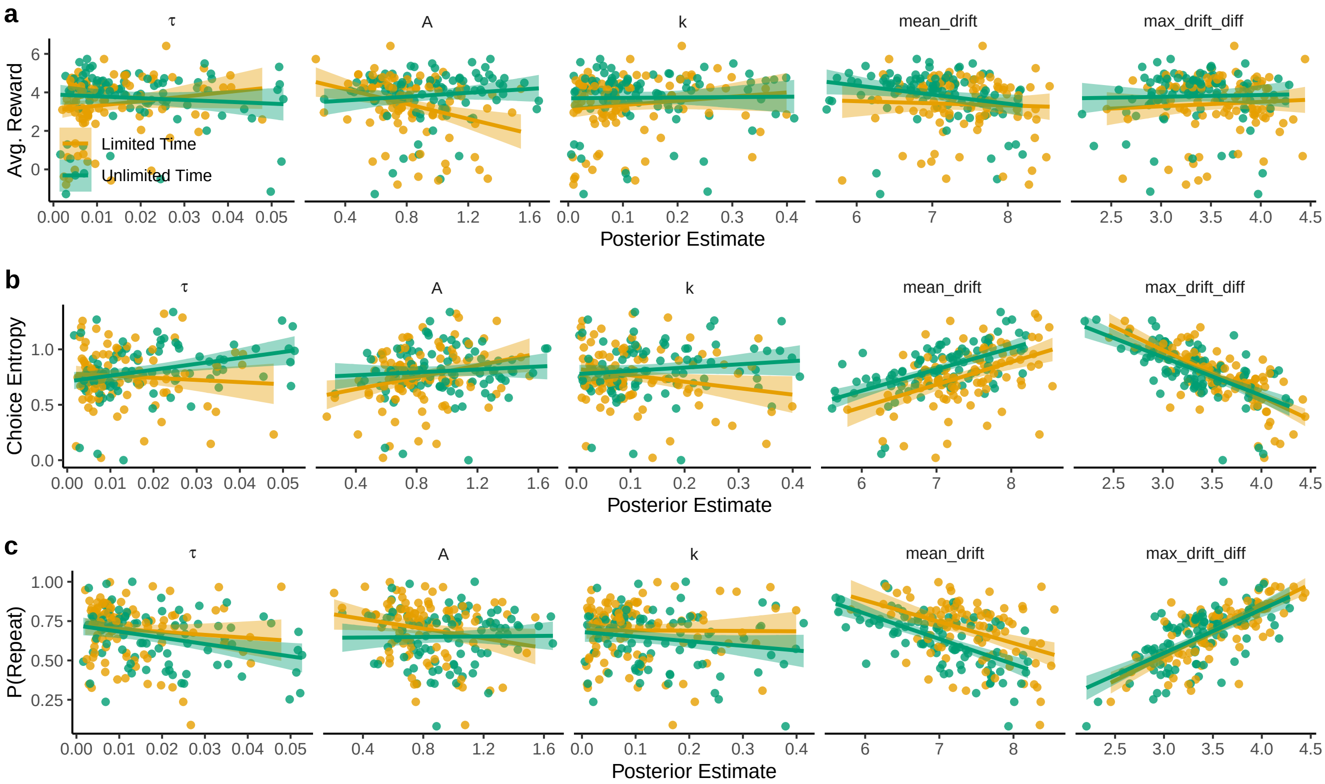

Figure S13. Comparison of LBA parameters and behavior. Each dot shows the mean posterior for each participant in each time condition, while the lines and ribbons are a linear regression and 95% CI. τ is the non-decision time, A is the maximum starting evidence, k is the relative threshold, mean_drift is the average drift rate across all four options $\frac{1}{4}\sum_j v_j$, and max_drift_diff is the largest pairwise difference in drift rates $\max_{i \neq j} |v_i - v_j|$. **a**) The only meaningful correlation between rewards and LBA parameters was found for maximum starting evidence A under time pressure ($r_\tau = -.25$, $p < .001$, $BF = 89$), where participants who were closer to making a decision prior to the start of a trial, earned lower payoffs. **b**) We find the strongest relationships between both drift rate variables and choice entropy, which were similar across time conditions. Participants with higher mean drift had more entropic choices (unlimited: $r_\tau = .37$, $p < .001$, $BF > 100$; limited: $r_\tau = .32$, $p < .001$, $BF > 100$), whereas participants with larger differences in drift rate were less entropic (unlimited: $r_\tau = -.49$, $p < .001$, $BF > 100$; limited: $r_\tau = -.57$, $p < .001$, $BF > 100$). We also find a weak correlation where higher maximum starting evidence was correlated with higher entropy for limited time rounds ($r_\tau = .15$, $p = .024$, $BF = 1.6$), and a moderate correlation where longer non-decision time corresponded to more entropic choices in unlimited time rounds ($r_\tau = .22$, $p = .002$, $BF = 18$). **c**) Similar to choice entropy, we again find the strongest relationship between the drift rate variables and the frequency of repeat choices, where higher mean drift produced less repeats (unlimited: $r_\tau = -.41$, $p < .001$, $BF > 100$; limited: $r_\tau = -.28$, $p < .001$, $BF > 100$), and larger differences in drift rate produced more repeat choices (unlimited: $r_\tau = .49$, $p < .001$, $BF > 100$; limited: $r_\tau = .58$, $p < .001$, $BF > 100$). We also find that higher starting evidence was correlated with more repeat choices in limited time rounds ($r_\tau = .58$, $p < .001$, $BF > 100$). In all plots, Tukey's fence has been applied to omit outliers for clearer visualizations, but all data are included in the statistical tests.

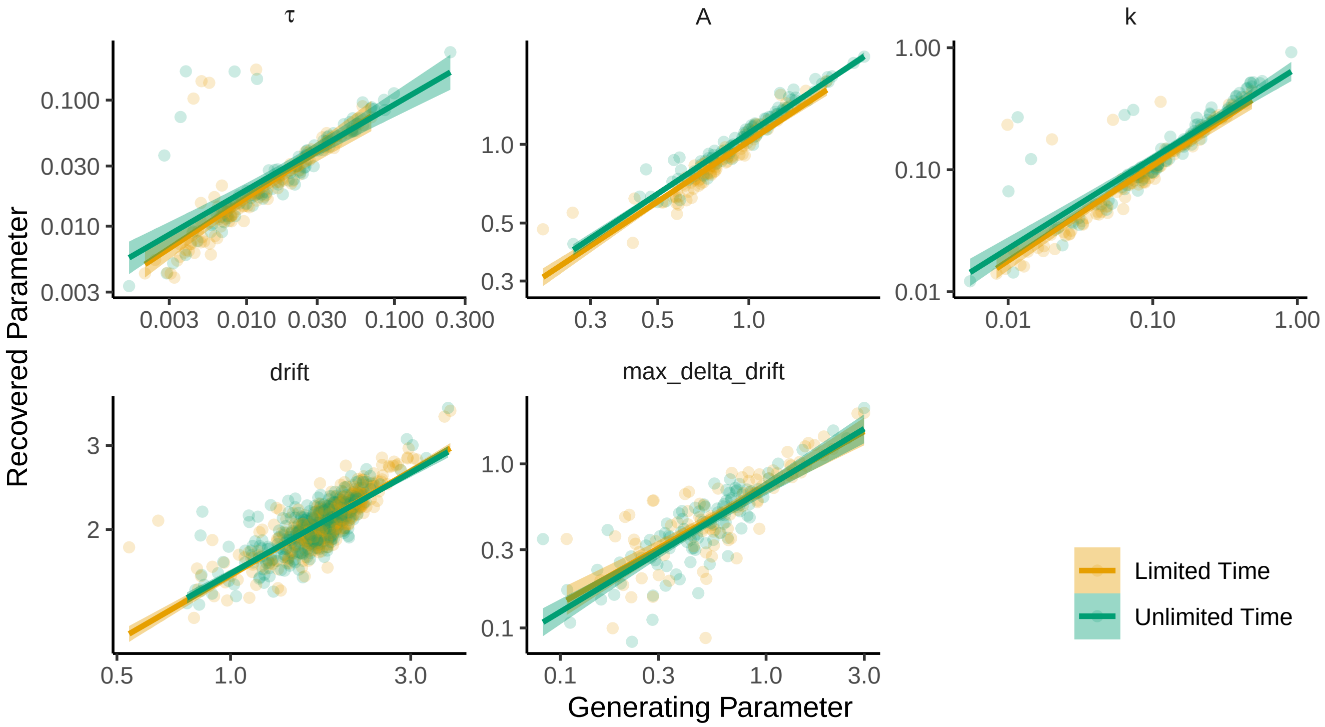

Figure S14. LBA recovery. Parameter recovery analysis, where the generating parameters on the x-axis were used to simulate participant choices and reaction times, upon which we used the same LBA estimation procedure and recovered the parameter estimates shown on the y-axis. Each dot is the posterior mean of a single participant (separated by time condition, but averaged across payoff conditions), with the line and ribbon showing a linear regression ± 95% CI. All parameters were recoverable in each time condition (all $r_\tau > .59$; $BF > 100$). τ is the non-decision time (unlimited time: $r_\tau = .76$, $p < .001$, $BF > 100$; limited time: $r_\tau = .76$, $p < .001$, $BF > 100$). A is the maximum starting evidence (unlimited time: $r_\tau = .88$, $p < .001$, $BF > 100$; limited time $r_\tau = .86$, $p < .001$, $BF > 100$). k is the relative threshold (unlimited time: $r_\tau = .84$, $p < .001$, $BF > 100$; limited time: $r_\tau = .84$, $p < .001$, $BF > 100$). Drift is the drift rate for each of the four options v_j (unlimited time: $r_\tau = .59$ $p < .001$, $BF > 100$; limited time: $r_\tau = .66$, $p < .001$, $BF > 100$). max_drift_diff is the largest pairwise difference in drift rates $\max_{i \neq j} |v_i - v_j|$ (unlimited time: $r_\tau = .64$, $p < .001$, $BF > 100$; limited time: $r_\tau = .63$, $p < .001$, $BF > 100$).

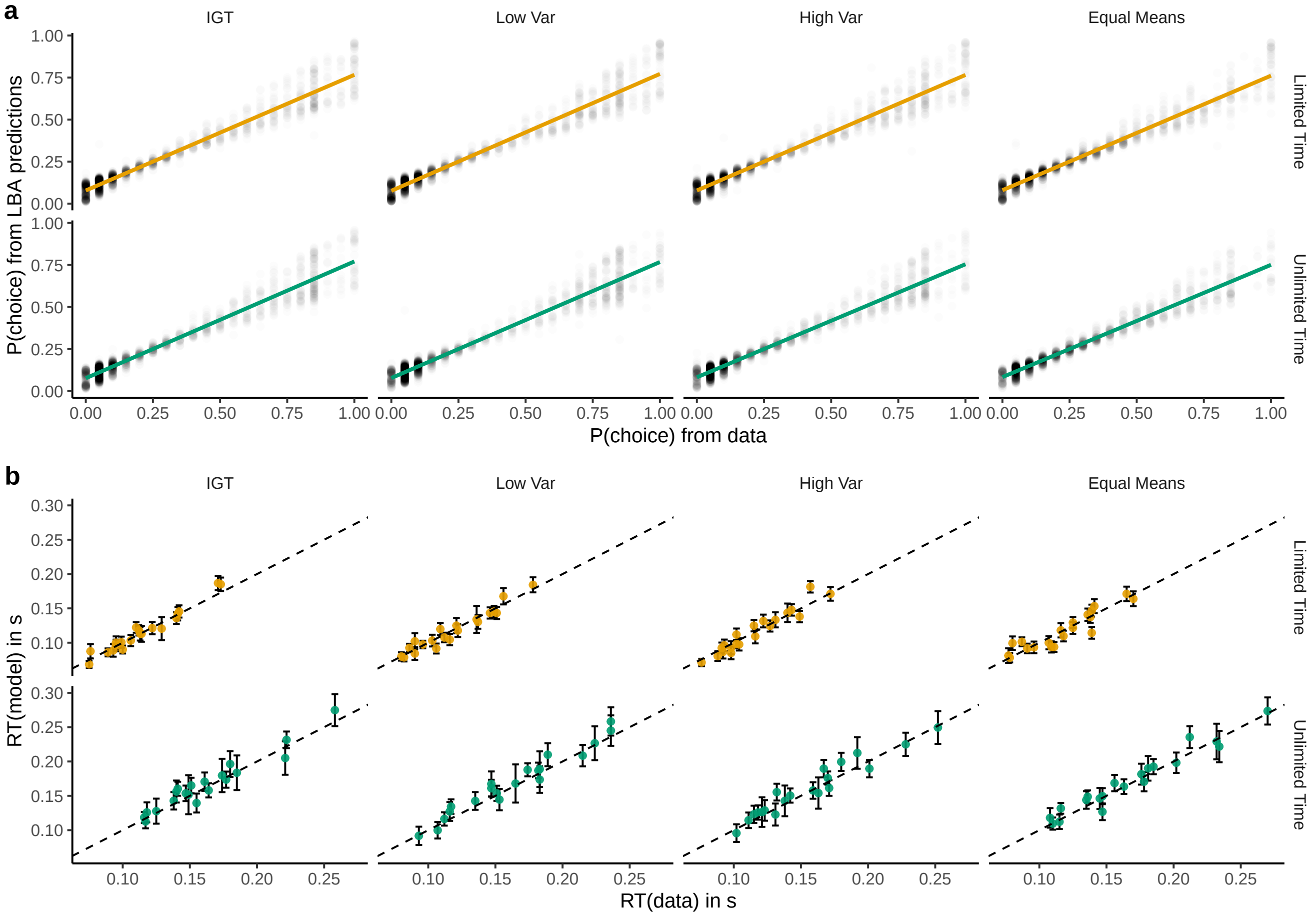

Figure S15. LBA simulations. **a**) Observed vs. simulated choice probabilities. For each set of participant parameter estimates, we generated 10k simulated choices that we used to define choice probabilities for each option (y-axis), which we compared against participants' observed choice probabilities (x-axis). Each dot represents the choice probabilities for each option for each set of parameter estimates. The colored line is a linear regression, with the ribbon showing the 95% CI. **b**) Observed vs. simulated RTs Using the same set of simulated data as above, we created a matched dataset where we sampled a simulated RT value yoked to each participant set of observed choices (i.e., an RT corresponding to the chosen option rather than any of the four options). We then aggregated the data by payoff condition and time pressure, and computed 20-quantiles along the participant RTs (x-axis), plotting the median (dot) and 95% CI (error bar) for the corresponding simulated RTs. The dashed line represents $y = x$.

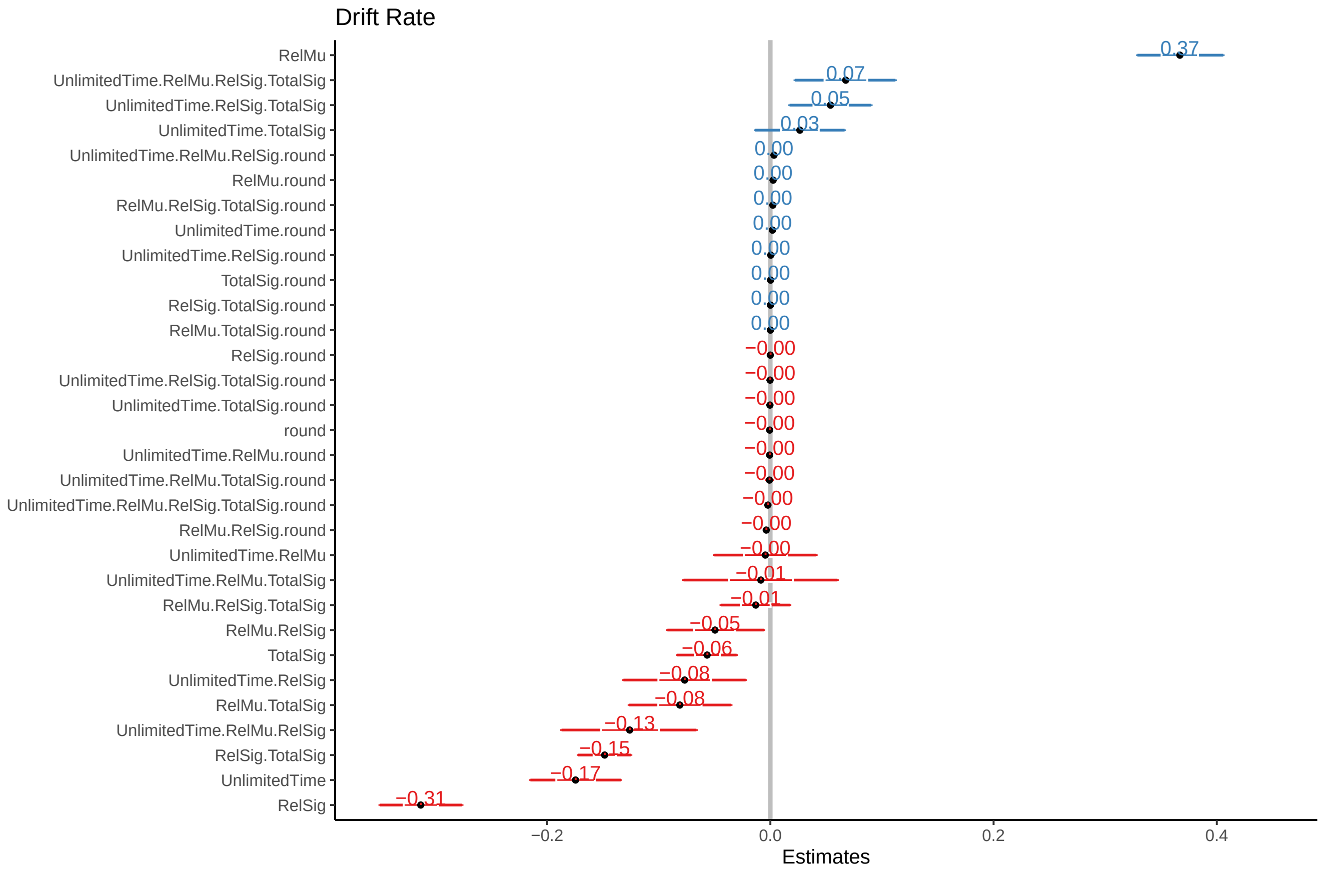

Figure S16. Coefficient plot of Drift Rate mixed effects regression. Posterior estimates of the Bayesian mixed effects regression predicting LBA drift rates. The mean posterior estimate is displayed numerically and indicated by the black dot, while the 95% HPD is illustrated by the length of the horizontal line. Coefficients are sorted by largest to smallest, with blue and red colors corresponding to estimates that are above or below 0, respectively, but do not indicate whether the difference is meaningful. See Figs. S17 for interaction plots. RelMu: relative reward; RelSig: relative uncertainty; TotalSig: total uncertainty.

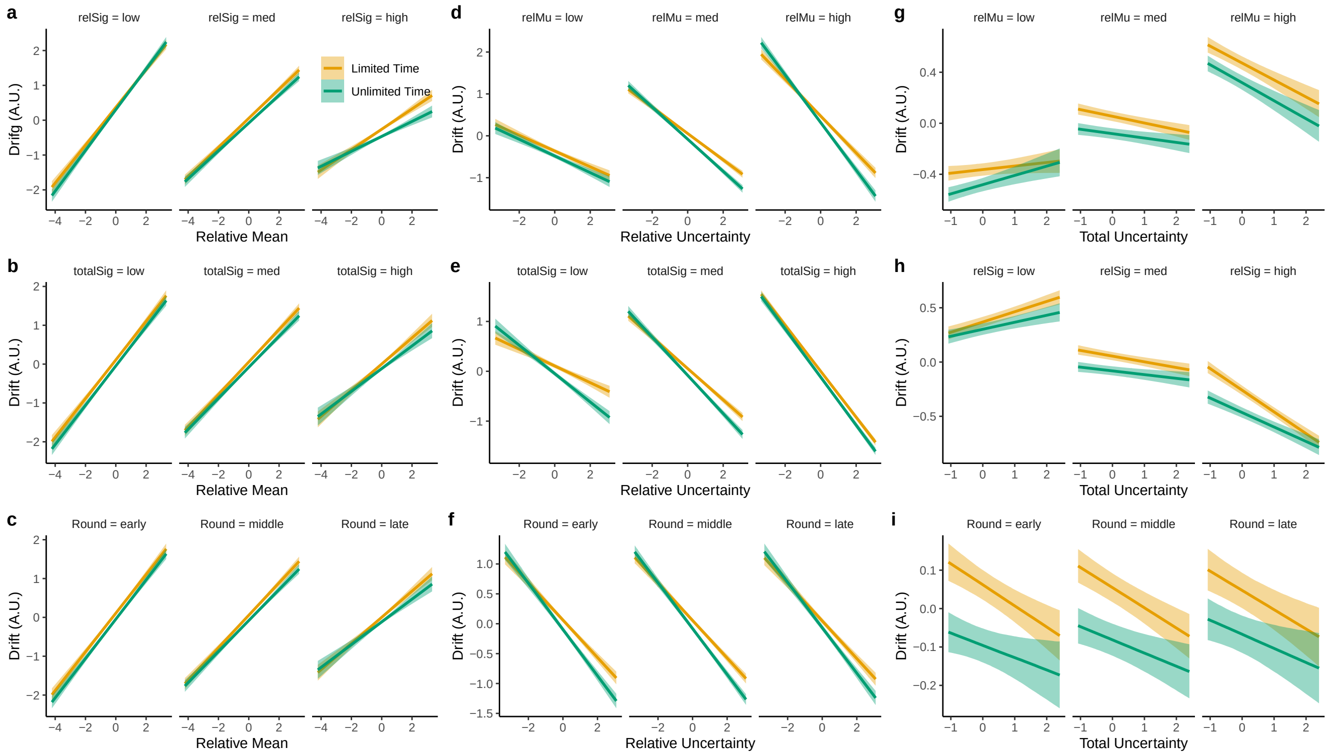

Figure S17. Marginal interaction plots for LBA mixed effects regression model. Marginal interactions of the LBA Bayesian mixed effects regression illustrated in Fig. S9. Interactions are grouped in terms of relative means (**a-c**), relative uncertainty (**d-f**), and total uncertainty (**g-i**). Continuous variables are split into discrete $[low, med, high]$ levels, based on $[mean - sd,\ mean,\ mean + sd]$. RelMu: relative reward; RelSig: relative uncertainty; TotalSig: total uncertainty.

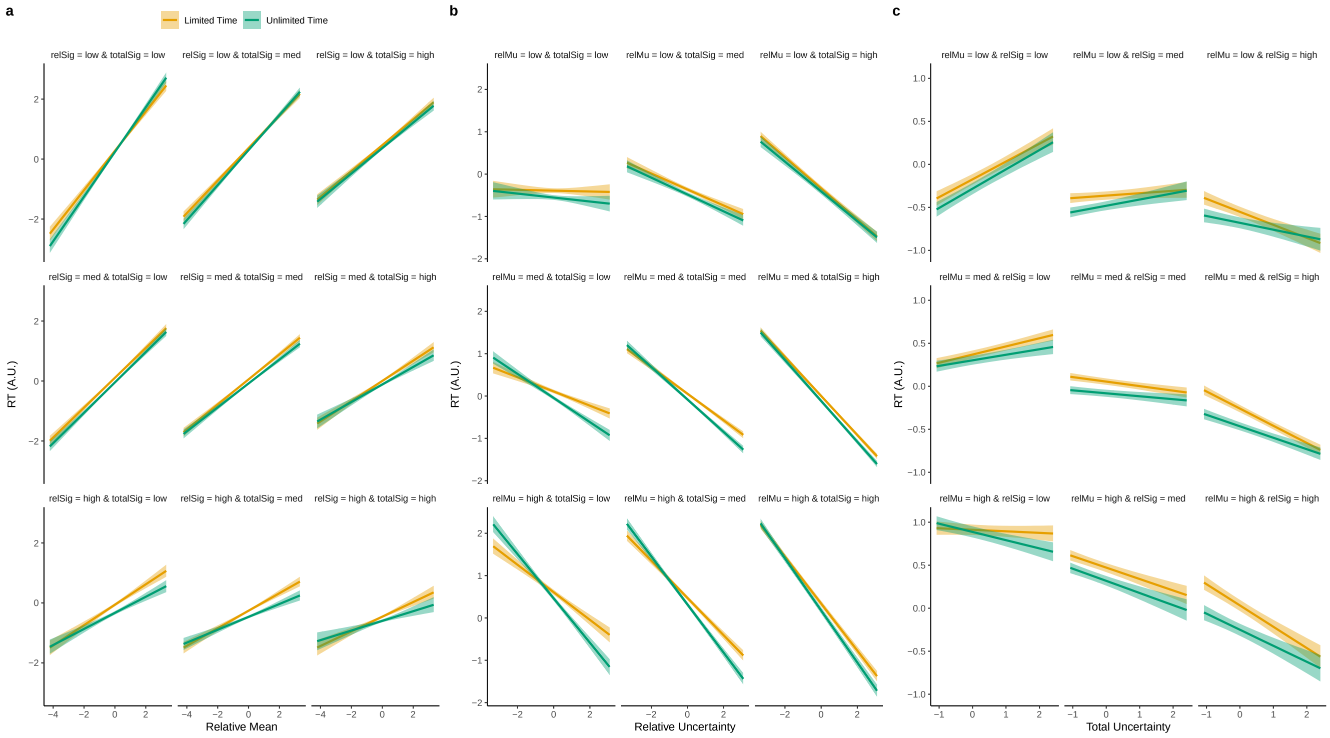

Figure S18. Four-way interactions for LBA mixed effects regression. Four-way interactions of the LBA Bayesian mixed effects regression illustrated in Fig. S16. Interactions are grouped in terms of relative means (**a**), relative uncertainty (**b**), and total uncertainty (**c**). Continuous variables are split into discrete $[low, med, high]$ levels, based on $[mean - sd,\ mean,\ mean + sd]$. RelMu: relative reward; RelSig: relative uncertainty; TotalSig: total uncertainty.

References

1. Rouder, J. N., Speckman, P. L., Sun, D., Morey, R. D. & Iverson, G. Bayesian t tests for accepting and rejecting the null hypothesis. *Psychon. Bull. & Rev.* **16**, 225–237 (2009).
2. Jeffreys, H. *The Theory of Probability* (Oxford, UK: Oxford University Press, 1961).
3. Ly, A., Verhagen, J. & Wagenmakers, E.-J. Harold jeffreys's default bayes factor hypothesis tests: Explanation, extension, and application in psychology. *J. Math. Psychol.* **72**, 19–32 (2016).
4. van Doorn, J., Ly, A., Marsman, M. & Wagenmakers, E.-J. Bayesian inference for kendall's rank correlation coefficient. *The Am. Stat.* **72**, 303–308 (2018).
5. Zellner, A. & Siow, A. Posterior odds ratios for selected regression hypotheses. In Bernardo, J. M., Lindley, D. V. & Smith, A. F. M. (eds.) *Bayesian Statistics: Proceedings of the First International Meeting held in Valencia (Spain)*, 585–603 (University of Valencia, 1980).
6. Jeffreys, H. An invariant form for the prior probability in estimation problems. *Proc. Royal Soc. Lond. Ser. A. Math. Phys. Sci.* **186**, 453–461 (1946).
7. Rouder, J. N., Morey, R. D., Speckman, P. L. & Province, J. M. Default bayes factors for anova designs. *J. Math. Psychol.* **56**, 356–374 (2012).
8. Gershman, S. J. Deconstructing the human algorithms for exploration. *Cogn.* **173**, 34–42 (2018).
9. Gelman, A. *et al.* *Bayesian data analysis* (CRC press, 2013).
